# An aldehyde-crosslinking mitochondrial probe for STED imaging in fixed cells

**DOI:** 10.1073/pnas.2317703121

**Published:** 2024-04-30

**Authors:** Jingting Chen, Till Stephan, Felix Gaedke, Tianyan Liu, Yiyan Li, Astrid Schauss, Peng Chen, Veronika Wulff, Stefan Jakobs, Christian Jüngst, Zhixing Chen

**Affiliations:** ^a^College of Future Technology, Institute of Molecular Medicine, National Biomedical Imaging Center, Beijing Key Laboratory of Cardiometabolic Molecular Medicine, Peking University, Beijing 100871, China; ^b^Department of NanoBiophotonics, Max Planck Institute for Multidisciplinary Sciences, Göttingen 37077, Germany; ^c^Clinic of Neurology, University Medical Center Göttingen, Göttingen 37075, Germany; ^d^Faculty of Mathematics and Natural Sciences, Cluster of Excellence Cellular Stress Responses in Aging-associated Diseases (CECAD), University of Cologne, Cologne 50931, Germany; ^e^Peking-Tsinghua Center for Life Science, Academy for Advanced Interdisciplinary Studies, Peking University, Beijing 100871, China; ^f^Peking University-Nanjing Institute of Translational Medicine, Nanjing 211800, China; ^g^Genvivo Biotech (PuHaiJingShan), Nanjing 211800, China; ^h^Fraunhofer Institute for Translational Medicine and Pharmacology Translational, Neuroinflammation and Automated Microscopy, Göttingen 37075, Germany; ^i^Cluster of Excellence “Multiscale Bioimaging: From Molecular Machines to Networks of Excitable Cells”, University of Göttingen, Göttingen 37099, Germany

**Keywords:** mitochondrial imaging, fixation, super-resolution imaging, CLEM

## Abstract

Fluorescence labeling of chemically fixed specimens, especially immunolabeling, plays a vital role in super-resolution imaging as it offers a convenient way to visualize cellular structures like mitochondria or the distribution of biomolecules with high detail. Despite the development of various distinct probes that enable super-resolved stimulated emission depletion (STED) imaging of mitochondria in live cells, most of these membrane-potential-dependent fluorophores cannot be retained well in mitochondria after chemical fixation. This lack of suitable mitochondrial probes has limited STED imaging of mitochondria to live cell samples. In this study, we introduce a mitochondria-specific probe, PK Mito Orange FX (PKMO FX), which features a fixation-driven cross-linking motif and accumulates in the mitochondrial inner membrane. It exhibits high fluorescence retention after chemical fixation and efficient depletion at 775 nm, enabling nanoscopic imaging both before and after aldehyde fixation. We demonstrate the compatibility of this probe with conventional immunolabeling and other strategies commonly used for fluorescence labeling of fixed samples. Moreover, we show that PKMO FX facilitates correlative super-resolution light and electron microscopy, enabling the correlation of multicolor fluorescence images and transmission EM images via the characteristic mitochondrial pattern. Our probe further expands the mitochondrial toolkit for multimodal microscopy at nanometer resolutions.

## Significance

Super-resolution stimulated emission depletion (STED) imaging of mitochondrial ultrastructure was mostly demonstrated with live cells due to the limited availability of fixable probes. The development of a mitochondrial probe that is both optically outstanding and retaining strong fluorescence signals after fixation would further democratize super-resolution imaging techniques for mitochondrial research. To address this challenge, we employ an emerging bioconjugation strategy to develop an aldehyde-fixable mitochondrial probe, PK Mito Orange FX (PKMO FX), which enables nanoscopic STED imaging both before and after fixation. With PKMO FX, we demonstrate multiplexed imaging of mitochondrial cristae and various cellular organelles, as well as correlative super-resolution light and electron microscopy.

Mitochondria are key organelles, which not only provide energy but also generate reactive oxygen species and regulate cell metabolism and apoptosis (1–3). Mitochondria-related research has become an active field at the forefront of life science research because structural and functional changes of mitochondria are closely related to various diseases (4, 5). Imaging is a key tool for studying mitochondria, including fluorescence microscopy (FM) and electron microscopy (EM) (6–8). FM, especially super-resolution fluorescence microscopy techniques such as stimulated emission depletion (STED) microscopy (9, 10), have enabled the visualization of the intricate mitochondrial ultrastructure and of mitochondrial proteins (11). EM, as a perfect complement to FM, can depict the structure of membranes and macromolecules in the cell with a resolution of less than 1 nm (12, 13).

Fixation is a widely used tool in both FM and EM imaging as it preserves cellular structures at a specific time point, allowing researchers to study structures that are too dynamic to characterize in live cells (14). Compared to live cells, fixed cells are accessible to a wide range of well-established labeling strategies, especially immunolabeling which allows the localization of multiple proteins of interest (POI), providing detailed information about different cellular structures or molecular interactions (15). In addition, the elimination of motion artifacts caused by cellular movements can support a more detailed visualization of subcellular structures or facilitate the recording of larger areas or volumes. Practically, fixed samples are more accessible to advanced super-resolution microscopes which may not be available in every laboratory or research facility.

**Scheme 1.**
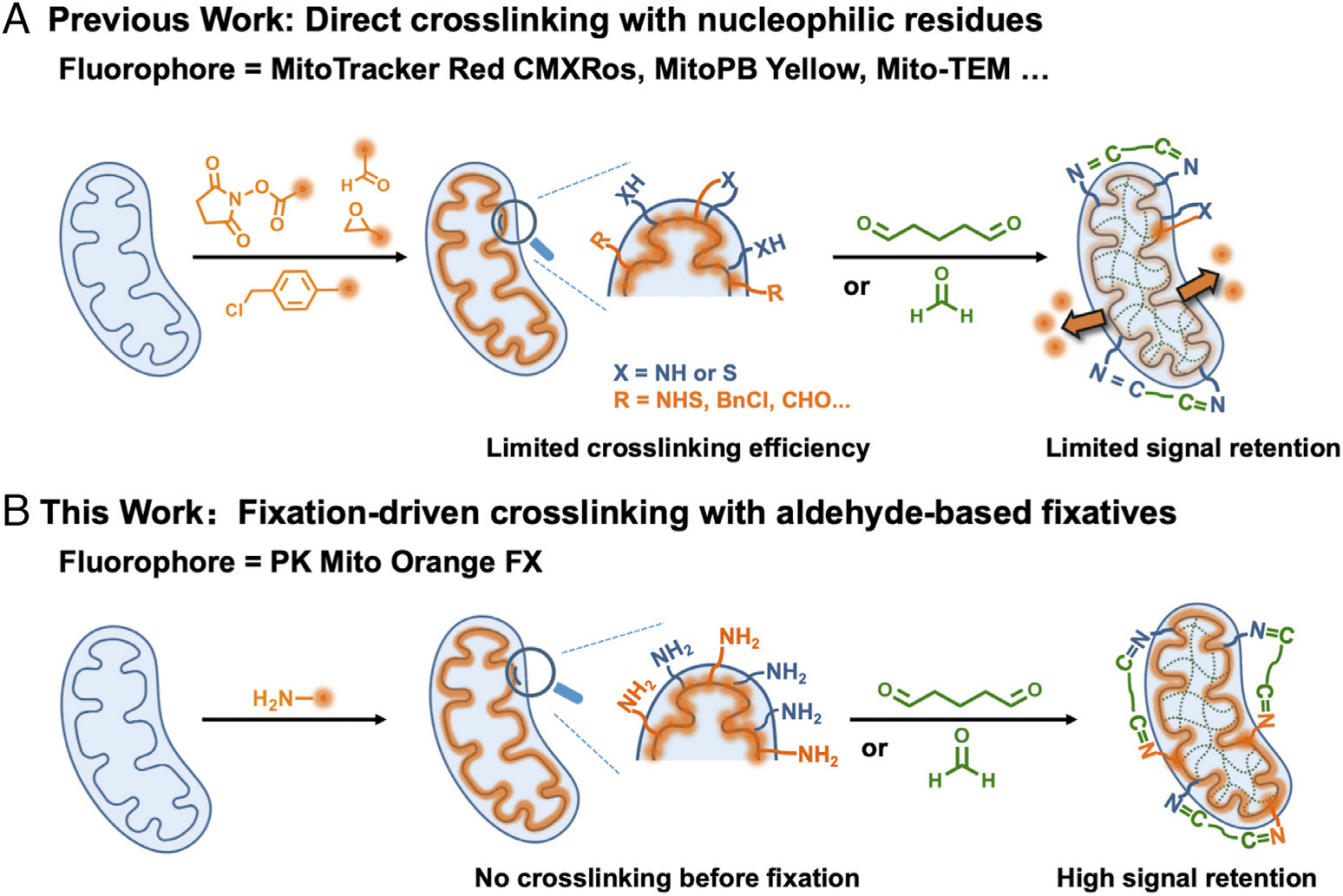
PK Mito FX probes exhibit high mitochondrial retention after aldehyde fixation. (*A*) Conventional reaction-based cationic mitochondrial dyes generally show poor fluorescence retention after fixation due to limited cross-linking efficiency. (*B*) The primary amino group of PK Mito Orange FX (PKMO FX) can react quickly with excess aldehydes-based fixatives before dissipating out of the mitochondrial cristae, ensuring high-density labeling of inner membrane.

In the field of mitochondrial nanoscopic imaging, fixable probes are paradoxically underdeveloped compared to their counterparts for live-cell imaging. For the imaging of mitochondria in fixed cells or tissues, an ideal probe should provide a specific and bright mitochondrial staining pattern and preferably enable super-resolved STED imaging of the mitochondrial ultrastructure. Additionally, it should retain high brightness and remain available for STED imaging after aldehyde fixation. Most existing mitochondrial probes rely on the mitochondrial membrane potential (MMP) to drive the hydrophobic cations specifically into the mitochondria (16, 17). Although well implemented in live cells, noncovalent mitochondrial probes will quickly diffuse and lose their localization when fixation causes the rapid dissipation of the MMP. A classical solution to this problem is to attach a reactive anchor to the probe that can react with nucleophilic residues of proteins, such as the thiol-reactive chlorobenzyl moiety in the MitoTracker series (18), the epoxide moiety in MitoPB Yellow (19), and the benzaldehyde moiety in mito-TEM (20). However, possibly due to the limited number of accessible nucleophiles on proteins, severe dye leakage and a significantly increased background often emerge after fixation (Scheme 1*A*). Therefore, although the mitotracker series dyes are widely used in confocal imaging and enabled pioneering post-fixation STORM imaging of mitochondria (21), super-resolved STED microscopy hinges on both advanced fluorophores and dense labeling to realize its full potential, placing higher demands on both the photophysics of fluorophores and the bioconjugation chemistry. Over the past years, a handful of mitochondrial probes for live-cell super-resolution microscopy has been developed (19, 22, 23), among which the PK Mito series stands out as gentle dyes for long time-lapse recordings with minimal phototoxicity (24, 25). However, currently available fixable mitochondrial dyes have not been successfully used to visualize fixed mitochondrial ultrastructure by STED super-resolution microscopy.

Herein, we tailor PK Mito Orange (PKMO) probe into its fixable version, PKMO FX, which enables STED imaging both before and after aldehyde fixation. Instead of introducing a reactive anchor that links the probe to mitochondrial proteins, we leverage a fixation-driven chemical cross-linking method that involves introducing a primary amine to facilitate efficient cross-linking between proteins, probes, and aldehyde fixatives (Scheme 1*B*). We demonstrate that upon aldehyde fixation, PKMO FX combined with metabolic labeling, chemigenetic labeling, or immunofluorescence allows multicolor STED microscopy of mitochondrial cristae, which also offers efficient labeling of the inner membrane for STED-CLEM workflows.

## Results

### Fixation-Driven Chemical Cross-Linking Enables Efficient Mitochondrial Fluorescence Signal Retention.

In our previous work, we investigated the properties of various fluorophores for live-cell STED imaging of mitochondria and found that Cy3.5-based PKMO offers excellent performance using STED microcopy with a 775 nm depletion laser (25). However, upon chemical fixation using aldehydes fixatives, it gradually diffuses into peripheral cellular membranes, especially the endoplasmic reticulum (*SI Appendix*, Fig. S1). To overcome this drawback, we intended to develop a fixable version of PKMO with post-fixation STED imaging potential based on the Cy3.5 chromophore. We initially conjugated traditional covalent reaction handles such as an N-Hydroxysuccinimide (NHS) ester or aldehyde moieties to Cy3.5, giving Cy3.5-NHS and Cy3.5-CHO respectively. Both dyes demonstrated promising live-cell staining. After chemical fixation, however, they exhibited a distinctly increased background and incompatibility with STED imaging (*SI Appendix*, Fig. S2). These findings suggest that while conjugation of reactive groups may facilitate fluorescence signal retention to some extent, there is still room for optimization to achieve long-term, stable, and efficient covalent fixation.

**Fig. 1.**
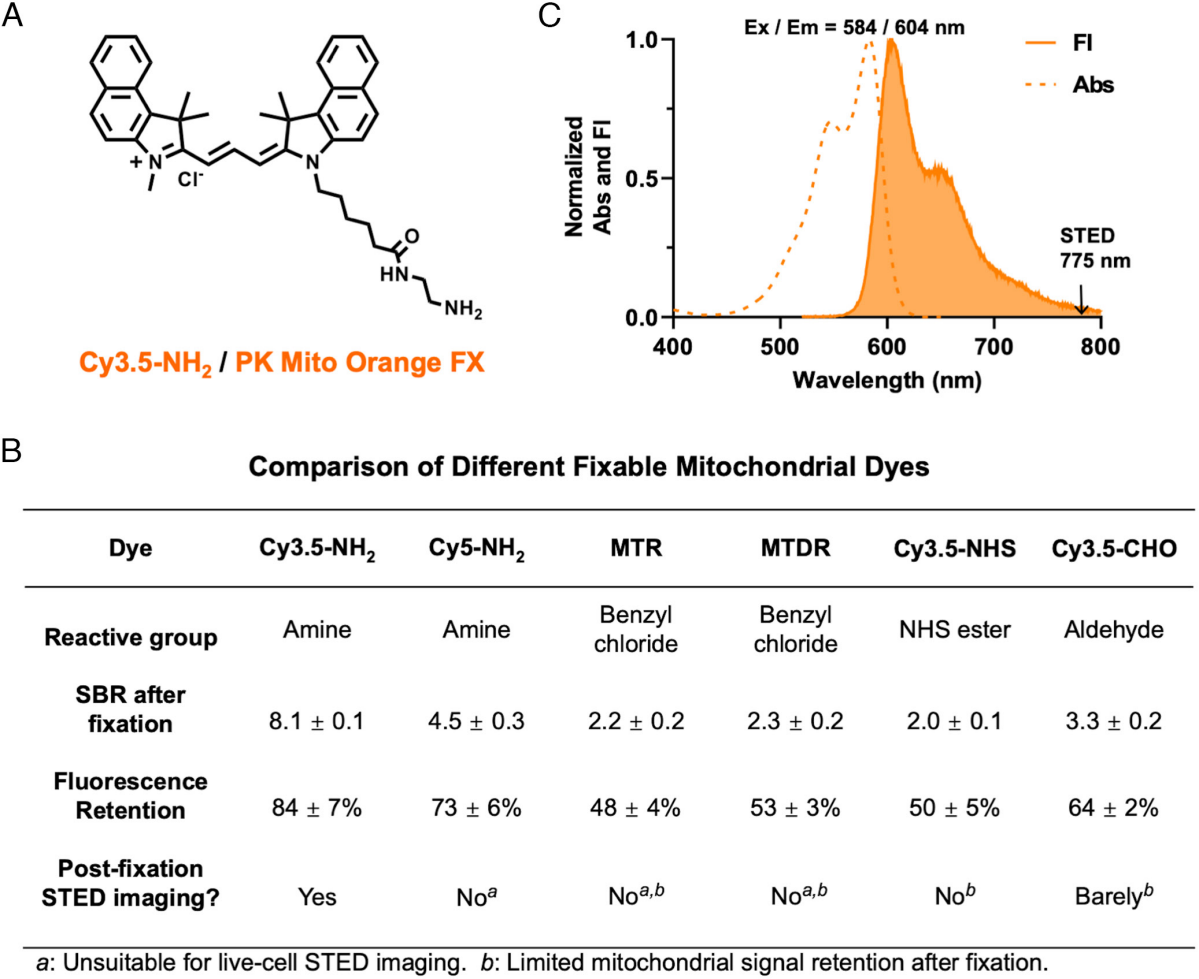
Fixation-driven chemical cross-linking strategy enables PKMO FX to achieve efficient fluorescence signal retention. (*A*) Chemical structure of Cy3.5-NH_2_ (PKMO FX). (*B*) Comparison of different fixable mitochondrial dyes after 2% GA fixation. Cy3.5-NH_2_ shows the highest Signal-to-background ratio (SBR) and mitochondrial fluorescence retention (MFR) after fixation. It has the best STED compatibility among commercial and designed mitochondrial dyes in this study. Abbreviations: MTR (MitoTracker Red CMXRos); MTDR (MitoTracker Deep Red FM). (*C*) Normalized absorption and fluorescence spectra of PKMO FX. Ex = 584 nm, Em = 604 nm.

Therefore, we turned to an alternative chemistry for fixation. Aldehyde-based chemical fixatives are broadly utilized in imaging of fixed cell, tissue, and complete organisms (26, 27). Glutaraldehyde (GA) and formaldehyde (FA) [commonly misreferred as paraformaldehyde (PFA)] can react with proximal amino acid side chains to form methylene bridges, thereby cross-linking proteins in situ (28). Inspired by this approach, we intended to take advantage of the reactivity of aldehydes and amines. We attached a terminal amino group to Cy3.5 and synthesized Cy3.5-NH_2_ (Fig. 1*A* and *SI Appendix*, Fig. S3). Similarly, we also synthesized Cy5-NH_2_ (*SI Appendix*, Fig. S4), which allowed for parallel comparison and may expand the spectrum of fixable dyes at our disposal. We hypothesized that these compounds could localize to mitochondria through charge interactions during the staining of live cells but achieve rapid condensation with excess aldehydes, preventing the excessive leakage of the fluorophore. This strategy using “fixation-driven chemical cross-linking” was recently demonstrated with immobilizing drug molecules in the brain for imaging their distribution (29).

To evaluate the retention of Cy3.5/Cy5-NH_2_, we compared the Signal-to-Background Ratio (SBR) and mitochondrial fluorescence retention (MFR) after GA fixation on confocal microscopy images with that of MTR, MTDR, Cy3.5-NHS, and Cy3.5-CHO (*SI Appendix*, Figs. S3–S6). To this end, HeLa cells were stained with each dye under optimized conditions and then fixed with 2% GA for 10 min. SBR quantifies the ratio between mitochondrial signal and background signal after fixation while the MFR tells the extent to which the fluorescence intensity of the mitochondria is preserved after fixation. The statistical results reveal that Cy3.5-NH_2_ achieves the best SBR (8.1) after fixation, whereas the SBR of MitoTracker fixable dyes is about only 2. More than 80% of the mitochondrial fluorescence signal could be preserved upon chemical fixation of Cy3.5-NH_2_, whereas the remaining mitochondrial fluorescence intensity was much weaker when using MTR (48%), MTDR (53%), and Cy3.5-NHS (50%) (Fig. 1*B*). Remarkably, Cy3.5-CHO exhibited a better SBR (3.3) and higher retention rate (64%) than benzyl chlorine-based and NHS-based dyes, highlighting the efficiency of the cross-linking reaction between amines and aldehydes. However, it is still inferior to Cy3.5-NH_2_, emphasizing the advantage of fixation-driven chemical cross-linking. The SBR (4.5) and retention rate of Cy5-NH_2_ (73%) were higher than MTDR, which proves the universality of our strategy in different chromophores.

Overall, Cy3.5-NH_2_ demonstrated the best SBR and highest MFR upon chemical fixation. Sharing similar chromophore structure, absorption, and emission spectra (Ex = 584 nm, Em = 604 nm) with the nonfixable PKMO (Fig. 1*C*), Cy3.5-NH_2_ is also well-tailored for STED nanoscopy using a 775 nm depletion laser and showed excellent performance in live cell imaging when recorded with commercial STED nanoscopes (Abberior Instruments STEDYCON, Leica TCS SP8 gSTED, *SI Appendix*, Fig S7). Hence, from here on, we refer to Cy3.5-NH_2_ as PKMO FX.

### PKMO FX Facilitates High-Resolution STED Imaging in Fixed Cells.

**Fig. 2.**
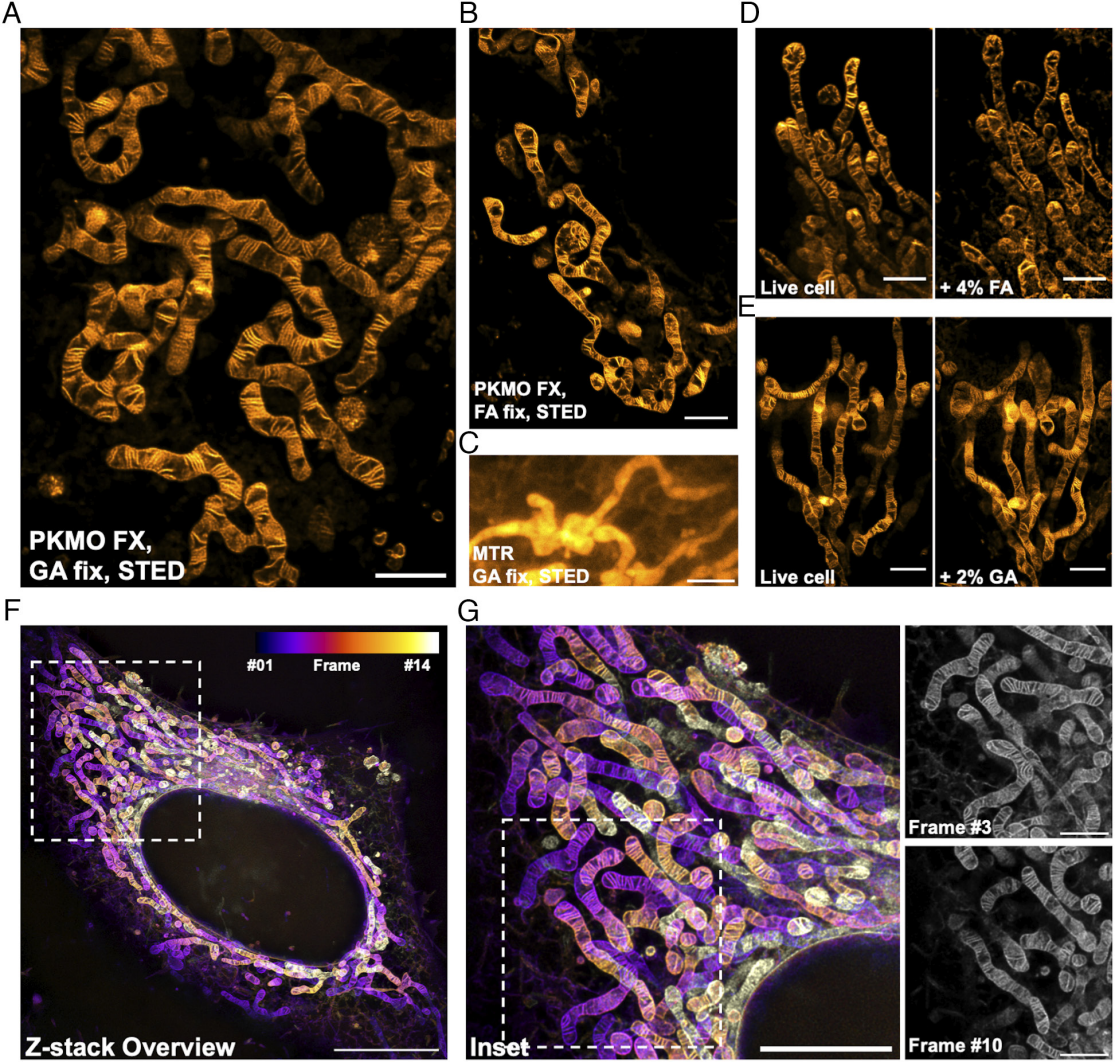
PKMO FX enables nanoscopic STED imaging of mitochondria in fixed cells. (*A*) STED imaging of mitochondrial cristae of a fixed COS-7 cell labeled with PKMO FX and fixed with 2.5% GA. (Scale bar, 2 μm.) (*B*) STED imaging of mitochondrial cristae of a fixed COS-7 cell labeled with PKMO FX and fixed with 4% FA. (Scale bar, 2 μm.) (*C*) STED imaging of mitochondria from a fixed HeLa cell labeled with MTR and fixed with 2.5% GA. (Scale bar, 2 μm.) (*D* and *E*) STED imaging of mitochondria labeled with PKMO FX in the same area before and after FA/GA fixation. After live cell imaging, 4% FA (*D*) or 2% GA (*E*) fixative was added to cells and STED images were acquired after 3 min. (Scale bar, 2 μm.) (*F*) Z-stack STED images of a HeLa cell labeled with PKMO FX and fixed with 2% GA. Color code image shows the whole-cell 3D reconstruction from 14 individual z-section images. Step size = 156 nm. (Scale bar, 10 µm.) (*G*, *Left*) Magnified images of the white boxed areas in *F*. (Scale bar, 5 µm.) *Right*: two close-up z-section images from frame #3 and #10. (Scale bar, 2 µm.)

To test the application of PKMO FX in post-fixation STED imaging, we stained COS-7 cells using 600 nM PKMO FX and 6 μM verapamil for 1 h. The addition of verapamil, an efflux pump inhibitor, is optional but can give more homogenous mitochondrial staining (30). This staining condition has been validated to be safe for maintaining mitochondrial morphology (*SI Appendix*, Fig. S8). Subsequently, the cells were washed and then fixed with 2.5% GA or 4% FA. We were able to capture the brightly labeled mitochondria and to resolve the mitochondrial cristae using STED microscopy after fixation (Fig. 2 *A* and *B*). After more than 10 d of fixation by GA, mitochondrial cristae stained by PKMO FX could still be clearly captured by STED microscopy, which proves that the aldehyde-induced cross-linking of PKMO FX is stable and allows long-term storage of specimens (*SI Appendix*, Fig. S9). In contrast to PKMO FX, MTR could hardly visualize clear mitochondrial cristae of live cells under the same STED imaging conditions (*SI Appendix*, Fig. S10). Additionally, the cristae became less resolvable after GA fixation due to the limited retention ability of MTR (Fig. 2*C*). The situation was even worse for MTDR, as the poor photostability of the chromophore Cy5 hinders its use in live-cell mitochondrial STED imaging (25), let alone in subsequent post-fixation imaging (*SI Appendix*, Fig. S11).

Chemical fixation of cells is known to be prone to fixation artifacts which are caused when the fixation process is too slow or by osmotic changes in the specimen (31, 32). To investigate the influence of FA/GA fixatives on mitochondria morphology, we performed STED nanoscopy of mitochondria before and after fixation, respectively. Both FA and GA fixation caused minor changes of the mitochondrial network morphology (Fig. 2 *D* and *E*). However, we occasionally observed swelling of mitochondria and noticeably altered crista-to-crista distances upon the fixation with 4% FA alone (*SI Appendix*, Fig. S12). This observation, which was less frequent in GA-fixed samples, is consistent with previous reports that FA fixation is more likely to introduce artifacts while GA largely preserves the ultrastructure by forming inter- and intramolecular covalent cross-linking faster and at longer distances (28, 33, 34).

Cristae in live mitochondria are highly dynamic and change their shape and orientation every few seconds (19, 22, 25, 35). Therefore, live-cell STED recordings have typically been restricted to the recording of single optical sections or small volumes to prevent motion artifacts (25). Fixed cells, however, allow longer acquisition times, facilitating the recording of larger volumes at greater detail. By employing PKMO FX staining and 2% GA fixation, we were able to record extended z-stacks to investigate the cristae architecture of whole cells (Fig. 2*F* and Movie S1). By imaging up to 14 individual optical sections at a step size of 156 nm, these recordings revealed the highly organized mitochondrial cristae (Fig. 2*G*) at an optical resolution of about 74 nm, which is very close to the resolution of live cristae (77 nm) (*SI Appendix*, Fig. S13). Thereby, PKMO FX allows us to analyze the fixed mitochondrial IM over large volumes at sub 100 nanometer resolution.

### PKMO FX Enables Analyzing the Mitochondrial Ultrastructure in Various Cell Types.

**Fig. 3.**
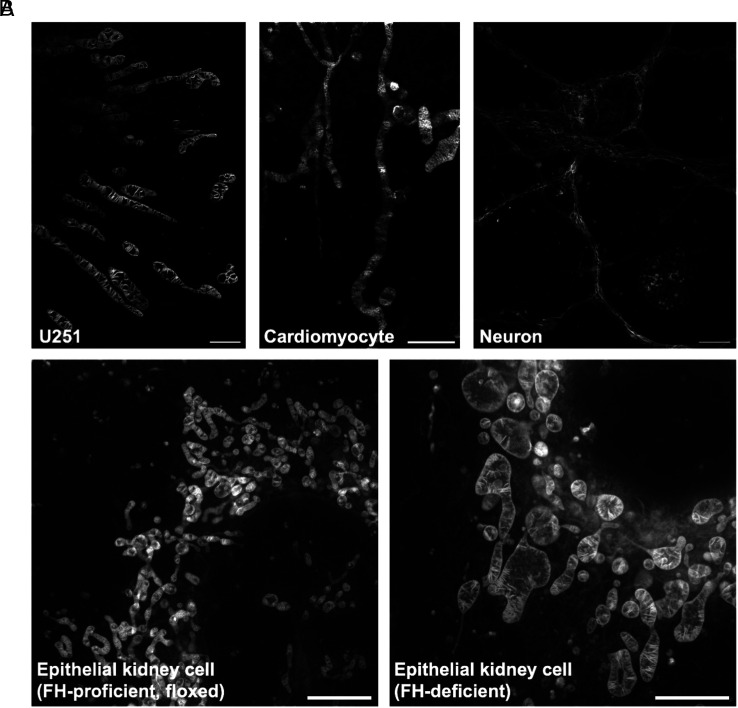
PKMO FX is a general mitochondrial probe for different cell lines. (*A*) PKMO FX-labeled mitochondria on different types of cells. From *Left* to *Right*: postfixation STED images of U251 cells, neonatal rat cardiomyocytes, and neuron cells. (Scale bars, 2 μm, 2 μm, 5 μm.) (*B*) STED images of FH-proficient (floxed) mouse epithelial kidney cells (*Left*) and FH-deficient cells (*Right*). In *Fh1*-KO cells, mitochondria show significantly swollen or swollen-elongated phenotypes with wider cristae spacing, uneven distribution of cristae, and the appearance of large hollow cavities compared to wild-type cells. (Scale bars, 5 µm.)

The spontaneous accumulation of PKMO FX inside the IM of mitochondria provides a simple approach for labeling various cell types and tissues without any genetic modification. We tested the staining of PKMO FX and subsequent fixation on several different cell lines, ranging from immortalized cells such as human glioblastoma astrocytoma (U251) cells to primary cells like neonatal rat cardiomyocytes and mouse hippocampal neurons. As expected, all cell lines showed bright labeling before and after fixation (Fig. 3*A*) and STED nanoscopy allowed us to compare the organization of the mitochondrial cristae. While U251 cells exhibited widely spaced lamellar cristae, the cristae in cardiomyocytes and neuron cells were more challenging to resolve, fully in line with their narrow spacing in these cell types (36, 37). PKMO FX labeling and subsequent STED microscopy also allowed us to detect structural changes caused by the deficiency of fumarate hydratase (FH), an enzyme involved in the tricarboxylic acid cycle which has recently been reported to be crucial for the remodeling of mitochondrial morphology (38, 39). STED nanoscopy recordings of mitochondria in wild type and FH-depleted mouse epithelial kidney cells were consistent with the structural changes previously reported by TEM (39) (Fig. 3*B*). While in FH-proficient (floxed) cells, mitochondria mainly exhibited a tubular morphology and densely stacked lamellar cristae, in *Fh1*-KO cells, we observed swollen mitochondria with wider cristae spacing, uneven distribution of cristae, and the appearance of large hollow cavities. Together, PKMO FX labeling and subsequent STED imaging can provide a reliable readout that partly supplant the EM protocol using a much simpler stain-fix-image protocol.

### PKMO FX Is Compatible with Fluorescent Proteins, Self-Labeling Tags, Click-Chemistry, and Immunolabeling.

**Fig. 4.**
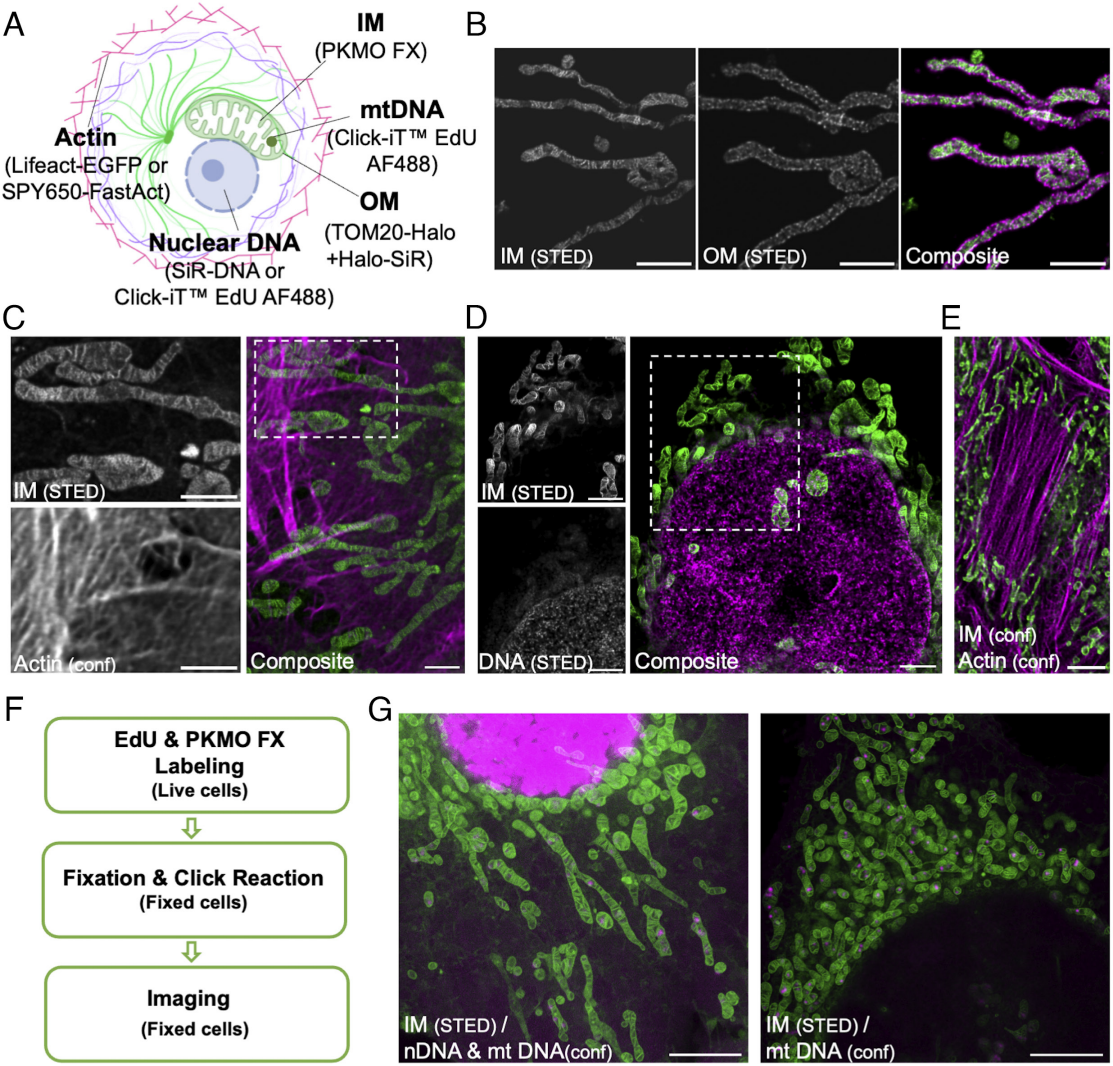
PKMO FX is compatible with various labeling strategies to achieve multicolor imaging of fixed cells. (*A*) Multicolor labeling strategies for imaging of mitochondrial subcompartments and related organelles. Abbreviations: IM (inner mitochondrial membrane); OM (outer mitochondrial membrane). (*B*) Two-color STED images of HeLa cells transfected with HaloTag-TOM20 and labeled with CA-SiR for OM and PKMO FX for IM. From *Left* to *Right*: split channels of IM, OM, and composite. (Scale bars, 2 μm.) (*C*) Two-color STED and confocal images of HeLa cells transfected with Lifeact-EGFP and stained with PKMO FX. *Left*: zoomed-in images of the white boxed areas of IM and actin. Actin was recorded in the confocal mode. *Right*: composite. (Scale bars, 2 μm.) (*D*) Two-color STED images of HeLa cells costained with PKMO FX and a fixable DNA probe: SiR-DNA. *Left*: zoomed-in images of the white boxed areas of IM and DNA. *Right*: composite. (Scale bars, 2 μm.) (*E*) Two-color confocal images of HeLa cells costained with PKMO FX and a fixable actin probe: SPY650-FastAct. (Scale bar, 5 μm.) (*F*) Flowchart showing the experimental procedure of the EdU-based metabolic labeling. (*G*) Two different cell states demonstrated by metabolic labeling. *Left*: cell cycle state I in which both nascent nuclear DNA (nDNA) and nascent mitochondrial DNA (mtDNA) are present. *Right*: cell cycle state II in which only nascent mtDNA spots are present. (Scale bars, 5 μm.)

Fluorescent proteins, self-labeling tags, and transfection-free fluorescent probes are popular labeling strategies for super-resolution fluorescence microscopy. We hypothesized that the staining and fixation protocol of PKMO FX should be compatible with such strategies, allowing multicolor recordings of fixed mitochondria. To this end, we labeled several cellular structures using different labeling strategies (Fig. 4*A*). First, we expressed the mitochondrial outer membrane (OM) protein TOM20 fused to HaloTag in HeLa cells and costained the cells with PKMO FX and the deep-red fluorophore chloroalkane-Silicon rhodamine (CA-SiR) (40). As CA-SiR binds covalently to the targeted protein and is efficiently depleted using the 775-nm STED beam, we were able to differentiate the PKMO FX-enriched inner membrane from the surrounding OM after chemical fixation (Fig. 4*B*). Similarly, HeLa cells expressing Lifeact-EGFP with PKMO FX provided two-color recordings of the actin cytoskeleton and the mitochondrial inner membrane (41) (Fig. 4*C*).

Next, we combined PKMO FX with fixable transfection-free probes for different cellular structures. Colabeling with SiR-Hoechst, a deep-red DNA dye suitable for super-resolution STED imaging (42), allowed us to obtain two-color STED recording of mitochondria and DNA in fixed cells (Fig. 4*D*). Similarly, the actin probe SPY650-FastAct (30) facilitated the recording of F-actin and mitochondria after costaining and fixation (Fig. 4*E*).

Metabolic labeling is a commonly used but more distinctive labeling strategy, which can provide valuable information about newly synthesized biomacromolecules. The thymine (T)-analog EdU can be incorporated into newly synthesized DNA by replacing thymine during cell proliferation and can be detected by a subsequent click reaction (43). For this, we incubated the cells with both EdU and PKMO FX and labeled the incorporated EdU after fixation using click chemistry (Fig. 4*F*). To avoid any negative effects of permeabilization agents on the staining brightness and ultrastructure, the permeabilization step used in typical click chemistry protocols was skipped (44). This strategy allowed us to investigate the mitochondrial ultrastructure, nuclear DNA, and mitochondrial DNA (mtDNA) simultaneously. After 2 h incubation of EdU, the nascent DNA could be detected in all cellular DNA or just in mtDNA depending on the different cell cycle states (Fig. 4*G* and Movie S2).

**Fig. 5.**
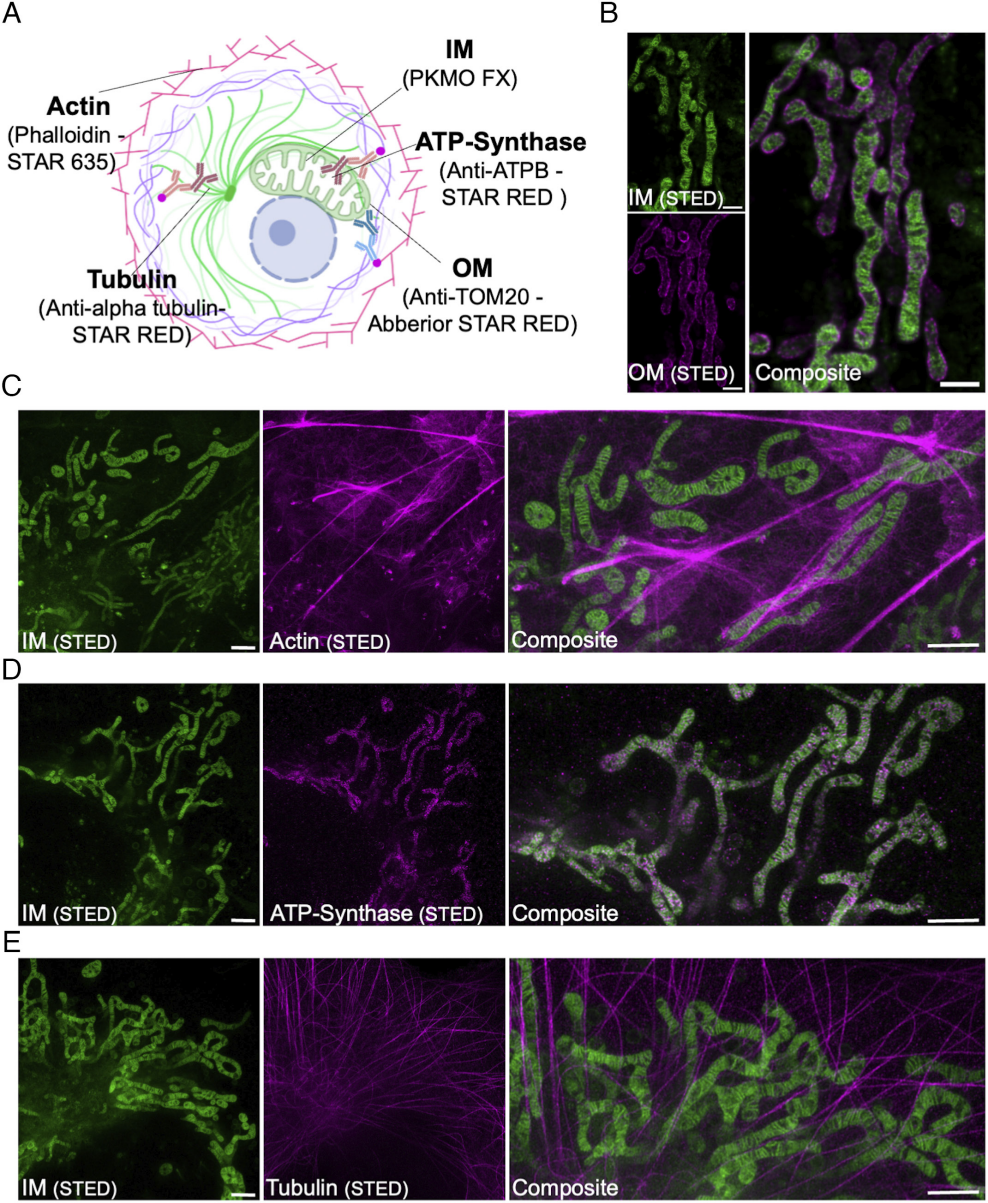
PKMO FX in conjunction with immunolabeling gives an alternative and convenient way for multicolor imaging. (*A*) Immunolabeling strategies for multicolor fixed cell imaging. (*B*) Two-color STED imaging of IM and OM in fixed HeLa cells. HeLa cells were first stained with PKMO FX and then fixed with 2% GA for 15 min before immunolabeling of Tom20. *Left*: split channels of IM and OM. *Right*: composite. (Scale bars, 1 μm.) (*C*–*E*) Two-color STED imaging of fixed COS-7 cells showing IM and actin, ATP-synthase, or tubulin, respectively. COS-7 cells were first stained with PKMO FX and then fixed with 2% GA for 20 s followed by 4% FA for 8 min before subsequent immunolabeling. *Left*: split channels of IM. *Middle*: split channels of POI. *Right*: Zoom-in views of composite. (Scale bars, 1 μm.)

Immunolabeling has been the most common approach for fluorescent labeling of specific proteins. It requires chemical fixation, followed by incubation with fluorescently labeled antibodies or toxins to label specific target molecules. In general, FA fixation is recommended in these protocols as GA fixation can hinder the molecules from reaching their binding site. A common strategy to counteract structural damage during FA fixation is the combination of FA and GA (31, 33, 34). We compared the postfixation SBR from confocal images and STED imaging performance of PKMO FX and MT dyes at 4% FA plus 0.1% GA fixation condition, which are commonly used in immunolabeling. As expected, PKMO FX outperformed MT dyes on both SBR and STED imaging performance (*SI Appendix*, Fig. S14). To determine the performance of PKMO FX in combination with a standard immunofluorescence protocol, we fixed PKMO FX-labeled cells with GA and FA and stained various proteins using antibodies or protein-binding toxins (Fig. 5*A*). Immunolabeling of TOM20 could still be achieved after fixation with 2% GA, which allowed us to visualize the OM and IM in fixed HeLa cells (Fig. 5*B*). Likewise, we were able to label mitochondria together with F-actin, the mitochondrial ATP synthase subunit b, or tubulin in COS-7 cells (Fig. 5 *C*–*E*), but the protocol of fixation underwent some specific optimization (*SI Appendix*, Fig. S15). The cells were first fixed with 2% GA for 20 s then replaced with 4% FA for 8 min. In summary, by combining immunolabeling with PKMO FX, the interactions between various cellular structures and mitochondria in the cell can be mapped in fine detail.

### PKMO FX Enables STED-CLEM and Two-Color CLEM.

FM is a powerful tool to identify specifically labeled biomolecules but generally lacks a holistic overview of cells. Electron microscopy, in contrast, shows all cellular components at unrivaled resolution but identifying specific biomolecules is technically demanding. These limitations can be overcome with correlated light and electron microscopy (CLEM), which combines the strengths of both approaches to provide “lighted-up ultrastructure” (45, 46). Although CLEM has flourished for decades, one of the key challenges in the field has remained finding ways to maintain high fluorescent labeling intensity after chemical fixation (47, 48) as well as precise merging of the two modes of images.

**Fig. 6.**
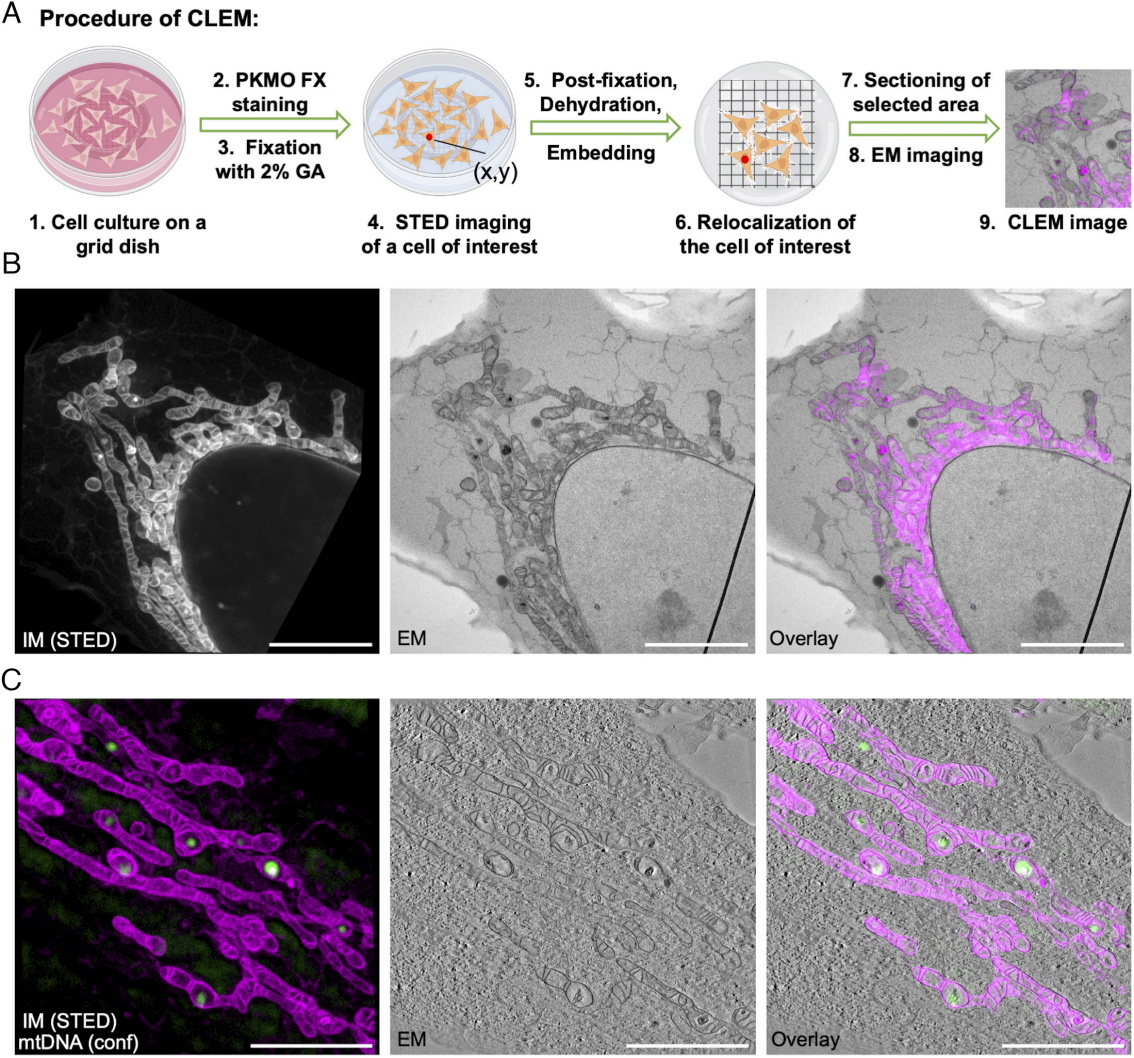
Multiplexed STED-CLEM using PKMO FX. (*A*) Experimental procedure of pre-embedding CLEM. (*B*) *Left*: STED image of a PKMO FX-labeled, 2% GA-fixed HeLa cell. *Middle*: TEM image of the same area. *Right*: Overlay of the FM and EM images. (Scale bars, 5 µm.) (*C*) Two-color CLEM experiment. *Left*: STED image of a PKMO FX-labeled, 2% GA-fixed HeLa cell (magenta). Background-subtracted confocal image of mtDNA (labeled with SYBR Gold, green). *Middle*: Single image extracted from an electron tomography stack. *Right*: Overlay of the two FM images with the EM image. (Scale bars, 3 µm.)

Considering its high brightness and superior ability to retain in mitochondria after chemical fixation, we hypothesized that PKMO FX would be an excellent reagent for CLEM. In general, CLEM can be applied as a pre- or post-embedding technique (45, 49, 50). Although CLEM is preferably performed directly on sections on the EM grid to achieve a perfect match between LM and EM, the OsO_4_ commonly used in postfixation and staining has strong oxidizing properties and can inevitably destroy the chromophore. As a result, post-embedding CLEM procedures are often incompatible with super-resolution FM (45, 51). Because of that, we developed a pre-embedding CLEM routine to enable STED-CLEM. HeLa cells were grown on a grid dish, labelled with PKMO FX, chemically fixed with GA, recorded by STED nanoscopy, and finally prepared for transmission EM. After embedding the cells in resin, we located the same cell by the coordinate information of the gridded dish and then sectioned it for EM imaging (Fig. 6*A*). With this approach, we were able to overlay STED and TEM images of the same mitochondria (Fig. 6*B*).

Technically, this approach is not limited to a single fluorescence channel. To demonstrate the feasibility of two-color CLEM, we combined mtDNA staining by SYBR Gold with PKMO FX staining (52). Using a CLEM protocol, we were able to visualize mtDNA spots with very high localization precision showing their distribution at the large ridge interval within the cristae (22) (Fig. 6*C*) which would not be possible by EM alone. Essentially, the correlation of mitochondrial signal in FM and EM generally bridges other fluorescence channels with EM images, especially those acquired with other synthetic dyes, fluorescent proteins or self-labeling tags which can circumvent the membrane-disruptive immunostaining protocols. Therefore, PKMO FX could help to generally improve CLEM experiments by acting as internal fiducial with very high localization precision.

## Conclusion and Discussion

In this study, we present PKMO FX, a fixable mitochondrial probe for STED nanoscopy and STED-CLEM. From the perspective of chemical probes, super-resolution imaging methods challenge both the optical properties and labeling efficiency of next-generation fluorescent markers. The fixation-driven chemical cross-linking strategy employed here basically addressed the leakage issue of potential-driven cationic mitochondrial stains, offering sufficient fluorophore density in the inner mitochondrial membrane for nanoscopy of mitochondrial cristae. This strategy underscores the power of advanced bioconjugation in synergy with fluorophore chemistry. Possible future directions include the following: 1) Fixable mitochondrial dye palette in different spectral regions; 2) Fixable mitochondrial dyes for nanoscopic methods based on single-molecule localization techniques (53, 54); 3) Next-generation dyes that are more cell permeable for faster stainings which can eventually be used in fixed or expanded tissues.

As a fixable mitochondrial dye, PKMO FX offers STED images of fixed cells whose quality is approaching those acquired in live cells, with only minor background staining. We also recapitulate that GA fixation gives minimal structural distortion to the delicate ultrastructure of mitochondria compared to FA fixation (55, 56). Moreover, optimized fixation protocol combining GA and FA can mostly preserve the membrane structure of mitochondria while at the same time enabling immunostainings. Bearing these new features, the PKMO FX probe will open exciting venues. 1) With the distinctive mitochondrial structures that are visible in both FM and EM channels, the CLEM protocol can now be upgraded to STED-CLEM. This offers a handy method to add one or even more molecular-specific information onto the global map of cells in EM. 2) Antibodies and metabolic labeling tools can now be easily added into the toolkit for mitochondrial investigations under nanoscopy, which is previously cumbersome due to the lack of a mitochondrial probe compatible with postfixation immunostaining or click-chemistry. 3) For practical considerations, fixed samples can be more easily transferred to imaging facilities equipped with advanced microscopes. Overall, we envision PKMO FX to cause another wave of infiltration of super-resolution imaging into general mitochondrial research.

## Materials and Methods

For a detailed description of the experimental procedures including chemical synthesis of PKMO FX, spectroscopy, cell culture, transfection, PKMO FX labeling, fixation and imaging, fluorescence retention assay, metabolic labeling, immunolabeling, CLEM (*SI Appendix*).

### Labeling of PKMO FX and Subsequent Fixation.

For optimal results, we recommend optimizing PKMO FX concentration and staining duration for each cell line. In general, for HeLa or COS-7 cells, we recommend staining the cells with 150 to 700 nM dye plus 6 to 10 μM verapamil for 15 min to 2 h. Verapamil is not essential for the staining, but 6 to 10 µM verapamil can be added to reduce heterogeneity of the staining. Cells were washed with fresh PBS buffer (pH = 7.4) twice after staining and fresh medium was added into the dish for live cell imaging. If the signal is noisy with low mitochondrial contrast, we recommend longer wash time in DMEM. After live cell imaging of PKMO FX, the cells were washed with fresh PBS buffer (pH = 7.4) three times and cells were fixed with preheated 2 to 2.5% GA or 4% FA for at least 10 min.

### EdU-based Metabolic Labeling.

For the metabolic labeling, the Click-iT Plus EdU Imaging Kit was used according to the manufacturer’s protocol but without using a permeabilization agent. HeLa cells were incubated with both PKMO FX (650 nM) and EdU (50 µM) for 2 h. Then, the cells were washed and fixed with 2% GA. The fixative was removed and the sample was washed twice with blocking solution (3% BSA in PBS). For EdU detection, the Click-iT Plus reaction cocktail including reaction buffer, copper protectant, Alexa Fluor 488 picolyl azide, and reaction buffer additive was freshly prepared and the sample was incubated for 30 min with this solution. The sample was washed three times with 3% BSA in PBS before imaging.

### Immunolabeling for ATPB and Tubulin.

COS-7 cells were stained with DMEM supplemented with 600 nM PKFO and 10 µM Verapamil for 2 h at 37 °C and 5% CO_2_. Before fixation, the cells were washed with prewarmed (37 °C) DMEM three times and then prefixed by immersion with prewarmed 2% GA in 0.1 M phosphate buffer (pH 7.4) for 20 s. The fixative was then replaced with prewarmed 4% FA in 0.1 M phosphate buffer (pH 7.4) and fixation was continued for 8 min at RT. The fixative was replaced by 0.1 M phosphate buffer and the cells were kept at RT for 10 min, followed by incubation with 0.1 M NH_4_Cl in 0.1 M phosphate buffer for 5 min. Cells were permeabilized with 0.25% Triton X-100 in 0.1 M for 5 min and washed five times with phosphate-buffered saline. All following steps were performed in PBS.

### Pre-embedding CLEM.

Cells were grown in glass-bottom dishes which were coated with a carbon finder pattern using a mask and a carbon coater ACE 200. After staining with 650 nM PKMO FX for 2 h, cells were fixed for 15 min at room temperature in 2% glutaraldehyde with 2.5 % sucrose and 3 mM CaCl_2_ in 0.1M HEPES buffer (pH = 7.4). Cells were washed three times with 0.1M HEPES buffer and fluorescent and brightfield images were taken using the SP8 gSTED microscope (Leica) with 100×/1.40 oil objective. Localization coordinates of cells of interest were noted.

Cells were incubated with 1% Osmium tetroxide and 1.5% potassium hexacyanoferrate for 30 min at 4 °C. After 3 × 5 min wash with 0.1M Cacodylate buffer, samples were dehydrated using ascending ethanol series (50%, 70%, 90%, 100%) for 7 min each at 4 °C. Cells were infiltrated with a mixture of 50% Epon/ethanol for 1 h, 66% Epon/ethanol for 2 h and with pure Epon overnight at 4 °C. TAAB capsules filled with Epon were placed upside down onto the glass bottom and cured for 48 h at 60 °C. Glass bottom was removed by alternatingly putting the dish into boiling water and liquid nitrogen. Block face was trimmed to the previous noted square using a razor blade and ultrathin sections of 70 nm or 300 nm for tomography were cut using an ultramicrotome and a diamond knife and stained with 1.5 % uranyl acetate for 15 min at 37 °C and 3% Reynolds lead citrate solution made from Lead (II) nitrate and tri-Sodium citrate dehydrate for 3 min.

Images were acquired using a JEM-2100 Plus Transmission Electron Microscope operating at 80 kV or at 200 kV for tomography equipped with a OneView 4K camera. Tomograms of 300 nm thick sections were generated using SerialEM (57) and IMOD (58). Overlay of TEM and STED images was generated using the EC-CLEM plugin (59) for the software ICY.

## Supplementary Material

Appendix 01 (PDF)

Movie S1. z-stack STED images of mitochondrial cristae in a HeLa cell labeled with PKMO FX and fixed with 2% GA. Scale bar = 10 μm.

Movie S2. Z-stack STED images of mitochondrial cristae (STED, grey) and nascent DNA (confocal, green) in a HeLa cell labeled with PKMO FX and Click-iT^TM^ AF488. Scale bar = 5μm.

## Data Availability

All study data are included in the article and/or supporting information. PKMO FX will be commercially available from Genvivo Biotech, Nanjing, China, and Spirochrome AG, Stein am Rhein, Switzerland.
