# Supplementary material for: An aldehyde-crosslinking mitochondrial probe for STED imaging in fixed cells
Source: Proc Natl Acad Sci U S A. 2024 Apr 30;121(19):e2317703121. doi: 10.1073/pnas.2317703121 (PMC11087744; doi:10.1073/pnas.2317703121)
Supplement: Supplementary file 1 — Appendix 01 (PDF) [file pnas.2317703121.sapp.pdf]

An Aldehyde-crosslinking Mitochondrial Probe for STED Imaging in Fixed Cells

Jingting Chen[1], Till Stephan[2,3], Felix Gaedke[4], Tianyan Liu[5], Yiyan Li[5], Astrid Schauss[4], Peng Chen[6,7], Veronika Wulff[4], Stefan Jakobs[2,3,8,9], Christian Jüngst[4*], Zhixing Chen[1,5,6,7*]

1. College of Future Technology, Institute of Molecular Medicine, National Biomedical Imaging Center, Beijing Key Laboratory of Cardiometabolic Molecular Medicine, Peking University, Beijing 100871, China
2. Department of NanoBiophotonics, Max Planck Institute for Multidisciplinary Sciences, Göttingen 37077, Germany
3. Clinic of Neurology, University Medical Center Göttingen, Göttingen 37075, Germany
4. University of Cologne, Faculty of Mathematics and Natural Sciences, Cluster of Excellence Cellular Stress Responses in Aging-associated Diseases (CECAD)
5. Peking-Tsinghua Center for Life Science, Academy for Advanced Interdisciplinary Studies, Peking University, Beijing 100871, China
6. PKU-Nanjing Institute of Translational Medicine, Nanjing 211800, China
7. GenVivo Tech, Nanjing 211800, China
8. Fraunhofer Institute for Translational Medicine and Pharmacology, Translational Neuroinflammation and Automated Microscopy, Göttingen 37075, Germany
9. Cluster of Excellence "Multiscale Bioimaging: from Molecular Machines to Networks of Excitable Cells", University of Göttingen, Göttingen 37099, Germany

To whom correspondence may be addressed: Christian Jüngst (cjuengst@uni-koeln.de), Zhixing Chen (zhixingchen@pku.edu.cn)

Table of contents

Materials and methods 1
Supplemental Figures 7
Chemical synthesis and characterization 14
Reference 21

Other supplementary materials for this manuscript include the following:

Movies S1 to S2

Legends for movies S1 and S2

Movie S1. z-stack STED images of mitochondrial cristae in a HeLa cell labeled with PKMO FX and fixed with 2% GA. Scale bar = 10 μm.

Movie S2. Z-stack STED images of mitochondrial cristae (STED, grey) and nascent DNA (confocal, green) in a HeLa cell labeled with PKMO FX and Click-iT ™ AF488. Scale bar = 5μm.

Materials and methods

Absorption and fluorescence spectroscopy

PKMO FX for spectroscopy were prepared as stock solutions (1 mM) in DMSO and diluted with acetonitrile (ACN) to 1 μM. Spectroscopy was performed using a 1cm square quartz cuvette. All measurements were taken at room temperature (25 ± 2 °C). Absorption and fluorescence emission spectra of the sample solution were measured using a Duetta spectrometer (HORIBA Instruments, Japan). Normalized spectra are shown for clarity.

Cell culture and transfection

The conditions of cell culture were slightly different as some experiments were carried out individually. Record them in detail as follows:

In post-fixation imaging; labeling of HaloTag, EGFP, and other fixable dyes; immunolabeling of TOM20 experiments shown in Fig 2a-e; Fig 3a; Fig 4 b-e; Fig 5b; Fig S1-S7a; Fig S9-S12, Fig S14-S15: HeLa or COS-7 cells were cultured in Dulbecco's modified Eagle's Medium (DMEM) (Gibco, 11965092; Thermo Fisher Scientific, Waltham, MA, USA) containing 10% (v/v) heat-inactivated fetal bovine serum (SE100-011; Vistech, Sydney, Australia) and 1% (v/v) penicillin sulfate and streptomycin (CC004; Macgene, Beijing, China) and incubated at 37 °C with 5% CO_2. Cells were plated in glass-bottom confocal dishes (Standard Imaging, China) for 24 h before staining and imaging. For transfection, cells were transfected transiently with corresponding plasmids using Lipo8000 reagent (Beyotime #C0533, China). After 6-12 h, residual transfection reagent was removed and the cells were washed with DMEM. The cells were maintained in fresh medium for another 18-24 h before staining with dyes for a designated duration.

In post-fixation z-stack STED imaging; imaging of mouse epithelial kidney cells; EdU-based metabolic labeling; CLEM experiments shown in Fig 2f-g; Fig 3b; Fig 4g; Fig 6 b-c; Fig S7b; Fig S8 and Fig S13: HeLa cells were cultured in DMEM (Gibco, 61965059, Thermo Fisher Scientific) containing 10% FBS (Gibco, 10270106, Thermo Fisher Scientific), 0.1% Pen/Strep (Gibco, 15140122, Thermo Fisher Scientific), 100 μM non essential amino acids MEM (Gibco, 11140035, Thermo Fisher Scientific), 1 mM Sodium Pyruvat (Gibco, 11360070, Thermo Fisher Scientific) and incubated at 37 °C with 5% CO_2. Cells were plated in glass-bottom confocal dishes (Ibidi, 81158, Germany) for 24 h before staining and imaging.

In immunolabeling of actin, ATP-synthase, and tubulin experiments shown in Fig 5c-e: COS-7 cells were cultured in DMEM medium with glutaMAX and 4.5 g/L glucose (Thermo Fisher Scientific, Waltham, MA, USA) at 37°C and 5% CO_2. The culture medium was supplemented with 100 U/ml penicillin and 100 μg/mL streptomycin (Merck Millipore, Burlington, MA, USA), 1 mM sodium pyruvate (Sigma-Aldrich, Munich, Germany), and 10% (v/v) fetal bovine serum (Merck Millipore). COS7-cells were seeded on 18 mm coverslips (Marienfeld GmbH & Co KG, Lauda-Königshofen, Germany, Cat. No. 0117580) and cultivated over night before imaging at 37°C and 5% CO_2.

PKMO FX labeling and fixation

For optimal results, we recommend optimizing PKMO FX concentration and staining duration for each cell line. In general, for HeLa or COS-7 cells, we recommend staining the cells with 100-700 nM PKMO FX for 15 min -2 h. Verapamil is not essential for the staining, but 6 -10 μM verapamil can be added to reduce heterogeneity of the staining. The cells were washed with fresh PBS buffer (pH =7.4) twice after staining and fresh medium was added into the dish for live-cell imaging. If the signal is noisy with dissatisfactory mitochondrial contrast, we recommend longer wash time in DMEM. After live-cell imaging of PKMO FX, the cells were washed with fresh preheated (37 °C) PBS buffer (pH =7.4) three times and fixed with preheated (37 °C) 2% - 2.5% GA or 4% FA for at least 10 min.

Specific staining conditions in this work:

Fig. 2a-b : 600 nM PKMO FX, 6 μM verapamil, 1 h.
Fig. 2c : 200 nM MTR, 20 min.
Fig. 2d-e : 650 nM PKMO FX, 8 μM verapamil, 1 h.
Fig. 2f-g : 650 nM PKMO FX, 10 μM verapamil, 2 h.
Fig. 3 : U251 cells: 600 nM PKMO FX, 1h. Neonatal rat cardiomyocytes: 600 nM PKMO FX, 1h. Neuron cells: 400 nM PKMO FX, 0.5 h. FH-proficient cells and FH-deficient cells: 650 nM PKMO FX, 10 μM verapamil, 2 h.
Fig. 4b : 600 nM PKMO FX, 200 nM Halo-SiR, 8 μM verapamil,1 h.
Fig. 4c-e : 600 nM PKMO FX, 8 μM verapamil,1 h.
Fig. 4g, Fig. 5, Fig. 6: Shown in the metabolic labeling, immunolabeling and CLEM parts respectively.

STED and confocal imaging

Partial images (Fig 2a-e; Fig 3a; Fig 4 b-e; Fig 5b; Fig S1-S7a; Fig S9-S12; Fig S14-S15) were taken on an Abberior STEDYCON microscope (Abberior Instruments GmbH, Göttingen, Germany) equipped with a 100×/1.45 oil objective (Nikon, Tokyo, Japan). PKMO FX was excited by a 561 nm laser and depleted with a 775 nm pulsed depletion laser with gating of 1-7 ns and dwell times of 10 μs. STED images (Fig 2 a,b,d,e; Fig 3 a; Fig 4 b-d; Fig 5b; Fig S7a, S12) were deconvolved using Huygens professional software.

The other part of imaging (Fig 2f-g; Fig 3b; Fig 4g; Fig 6b-c; Fig S7b; Fig S8 and Fig S13) was done on a Leica TCS SP8 gSTED microscope equipped with a 100×/1.40 oil objective. PKMO FX was excited at 594 nm laser using a white light laser and depleted with a 775 nm STED laser and time gating was applied (0.5-6 ns). Part of these STED images (Fig 2f, g; Fig 4g) were deconvolved using the internal Lightning deconvolution from Leica.

Another part of STED imaging (Fig 5c-e) were taken on an Expert Line dual-color STED 775 QUAD scanning microscope (Abberior Instruments). The microscope was equipped with a UPlanSApo 100×/1.40 Oil [infinity]/0.17/FN26.5 objective (Olympus). PKMO FX was excited by a 561 nm laser and depleted with a 775 nm STED laser. Deconvolution was done using Abberior Imspector software.

Measure of fluorescence retention ratio of different mitochondrial dyes after fixation

To simulate the real situation in actual use, HeLa cells were stained with the optimal or recommended staining conditions for each dye:

1. Cy3.5-NH_2 (PKMO FX): 600 nM plus 8 μM verapamil for 80 min.
2. Cy5-NH_2: 500 nM plus 8 μM verapamil for 60 min.
3. Mito Tracker Red: 200 nM for 20 min.
4. Mito Tracker Deep Red: 200 nM for 20 min.
5. Cy3.5-NHS: 600 nM plus 8 μM verapamil for 60 min.
6. Cy3.5-CHO: 1 μM plus 8 μM verapamil for 60 min.

After staining, live cell imaging was performed in one selected area (80 × 90 μm, contains about 4 -7 cells) under a 100 × confocal microscope. Then the cells were fixed with GA (final concentration was 2%) without moving the dish. The fixed cells of the same area were imaged 10 min after fixation. The laser intensity was consistent with that of live cell imaging. For one dye, the experiment was repeated three times. For each image, mitochondria were first sorted out by ImageJ: Image- Adjust- Threshold function, then the fluorescence intensity of mitochondria was counted. The fluorescence retention ratio is calculated as: fixed-cell mitochondrial intensity / living-cell mitochondrial intensity.

EdU-based metabolic labeling

For the metabolic labeling, the Click-iT Plus EdU Imaging Kit (C10337, Thermo Fisher Scientific) was used according to the manufacturer protocol but without using a permeabilization agent. HeLa cells were incubated with both PKMO FX (600 nM plus 10 μM verapamil) and EdU (50 μM) for 2 hours. After that the cells were washed with PBS buffer three times and fixed with 2% GA. The fixative was removed and the sample was washed twice with blocking solution (3% BSA in PBS). For EdU detection, the Click-iT Plus reaction cocktail including reaction buffer, copper protectant, Alexa Fluor 488 picolyl azide, and reaction buffer additive was freshly prepared and the sample was incubated for 30 minutes with this solution. Sample was washed several times with 3% BSA in PBS before imaging.

Immunolabeling

Immunolabeling for TOM20: HeLa cells were stained with DMEM supplemented with 650 nM PKFO and 10 μM Verapamil for 1 h at 37°C and 5% CO_2. Before fixation, cells were washed with pre-warmed (37 °C) DMEM three times and then prefixed by immersion with pre-warmed 2% GA in PBS buffer (pH 7.4) for 15 min. The fixative was washed by PBS buffer three times, then treated the cells with 0.5% Triton X-100 and 5% BSA in PBS buffer for 1 h. All following steps were performed in PBS. Cells were then incubated with rabbit-derived primary antibodies anti-TOM20 (ABclonal, Cat. No. A19403, 1:75) for 1 h at RT, followed by washed with PBS buffer for 5 min x 3 times. Then the cells were incubated with secondary antibodies (goat anti-rabbit) with abberior STAR RED fluorophore (Abberior, Göttingen, Germany, STRED-1002, 1:75) for 1h. Samples were washed 5 min x 3 times, post-fixed with 2% GA, mounted in PBS buffer and recorded under STED microscope the same day.

Immunolabeling for Actin, ATPB, and Tubulin: COS-7 cells were stained with DMEM supplemented with 600 nM PKFO and 10 μM Verapamil for 2 h at 37°C and 5% CO_2. Before

fixation, cells were washed with pre-warmed (37 °C) DMEM three times and then prefixed by immersion with pre-warmed 2% glutaraldehyde (Agar Scientific, Stansted, UK, Cat. No. AGR1020) in 0.1 M phosphate buffer (pH 7.4) for 20 sec. The fixative was then replaced with pre-warmed 4% formaldehyde (thermo scientific, Rockford, IL, USA, Cat. No. 28908) in 0.1 M phosphate buffer (pH 7.4) and fixation was continued for 8 min at RT. The fixative was replaced by 0.1 M phosphate buffer and the cells were kept at RT for 10 min, followed by incubation with 0.1 M NH_4Cl in 0.1 M phosphate buffer for 5 min. Cells were permeabilized with 0.25% Triton X-100 in 0.1 M phosphate buffer for 5 min and washed 5 times with phosphate buffered saline (PBS, 137 mM NaCl, 2.68 mM KCl, and 10 mM Na_2HPO_4, pH 7.4). All following steps were performed in PBS.

For labeling of F-actin, cells were incubated with Phalloidin-Abberior STAR 635 (Abberior GmbH, Göttingen, Germany, Cat. No. ST635-0100-20UG, 1:50) for 2h at RT. Labeling of microtubules and mitochondria was achieved by incubation with primary antibodies against alpha-tubulin (Merck, Cat. No. T6074-100UL, 1:700) and ATPB (Abcam, Cambridge, UK, Cat. No. ab5432, 1:100) for 1h at RT. Cells were washed five times and primary antibodies were detected by incubation with secondary sheep-anti-mouse antibodies (Jackson Immuno Research Laboratories, West Grove, PA, USA) custom-labeled with Abberior STAR RED NHS-ester (Abberior GmbH) for 1h at RT. Samples were washed 5 times, mounted in PBS or mounting medium (Ibidi GmBH, Gräfelfing, Germany, Cat. No. 50001) and recorded the same day.

Pre-embedding CLEM

Cells were grown in glass bottom dishes (MatTek, # P356-1.5-14-C) which were coated with a carbon finder pattern using a mask (Leica, # 16770162) and a carbon coater ACE 200 (Leica). Cells were stained with 650 nM PKMO FX plus 8 μM verapamil for 1.5 h. Cells were fixed for 15 min at room temperature in 2% glutaraldehyde (Sigma, # G5882-100ML) with 2.5 % sucrose (Roth, # 4621.1) and 3mM $CaCl_2$ (Sigma, # C7902-500G) in 0.1M HEPES buffer pH 7.4 (Roth, # 9105.1). Cells were washed three times with 0.1M HEPES buffer and fluorescent and brightfield images were taken using the SP8 gSTED microscope (Leica) with 100x/1.40 oil objective. Localization coordinates of cells of interest were noted.

Cells were incubated with 1% Osmium tetroxide (Science Services, # E19190) and 1.5% potassium hexacyanoferrate (Sigma, # P8131) for 30 min at 4°C. After 3 × 5min wash with 0.1M Cacodylate buffer (Applichem, # A21400100), samples were dehydrated using ascending ethanol series (50%, 70%, 90%, 100%) (VWR, # 153386F) for 7 min each at 4°C. Cells were infiltrated with a mixture of 50% Epon/ethanol for 1 h, 66% Epon/ethanol for 2 h and with pure Epon (Sigma, # 45359-1EA-F) overnight at 4°C. TAAB capsules filled with Epon were placed upside down onto the glass bottom and cured for 48 h at 60°C. Glass bottom was removed by alternatingly putting the dish into boiling water and liquid nitrogen. Block face was trimmed to the previous noted square using a razor blade and ultrathin sections of 70 nm or 300 nm for tomography were cut using an ultramicrotome (Leica Microsystems, UC6) and a diamond knife (Science Services # DU3530) and stained with 1.5 % uranyl acetate (Agar Scientific, # R1260A) for 15 min at 37°C and 3% Reynolds lead citrate solution made from Lead (II) nitrate (Roth, # HN32.1) and tri-Sodium citrate dehydrate (Roth #4088.3) for 3 min.

Images were acquired using a JEM-2100 Plus Transmission Electron Microscope (JEOL) operating at 80kV or at 200kV for tomography equipped with a OneView 4K camera (Gatan). Tomograms of 300nm thick sections were generated using SerialEM(1)and IMOD(2). Overlay of TEM and STED images were generated using the EC-CLEM plugin (3) for the software ICY.

Supplemental Figures

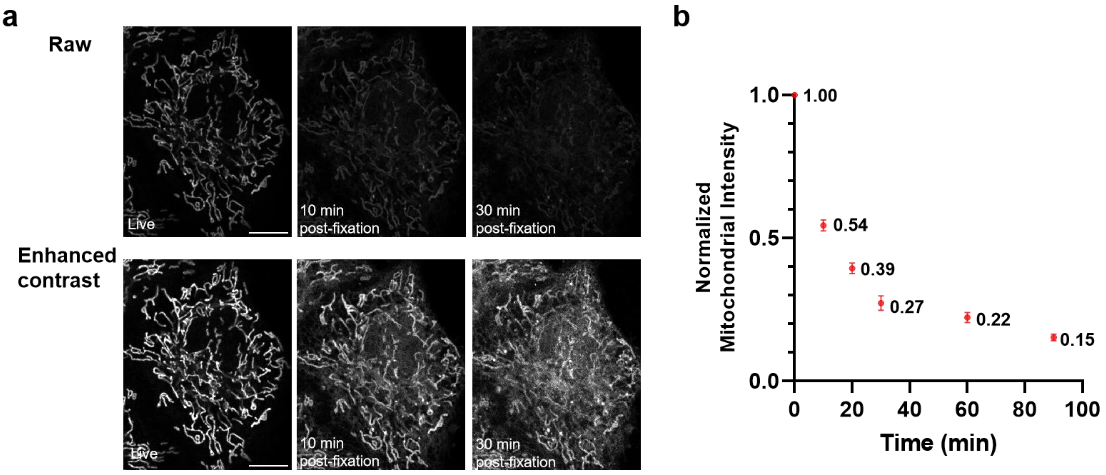

Fig S1. The non-covalent mitochondrial dye PKMO gradually dissipates from mitochondria after fixation.

PKMO exhibited a gradual leakage within 1 h of fixation. HeLa cells were stained with 400 nM PKMO for 20 min, and then fixed with 2% GA. The fixed cells were imaged at 0, 10, 20, 30, 60 and 90 min after fixation.

a. Raw (top row) and enhanced-contrast (bottom row) confocal images of HeLa cells. From left to right: live cells, 10 min post-fixation, 30 min post-fixation. Scale bars = 10 μm.

b. Statistics of mitochondrial intensity at different time points after fixation. The mitochondrial intensity of live cells (also recorded as 0 min after fixation) was normalized to 1.00. The mitochondrial intensity of fixed cells at 10, 20, 30, 60 and 90 min post-fixation is: 0.54, 0.39, 0.27, 0.22, 0.15.

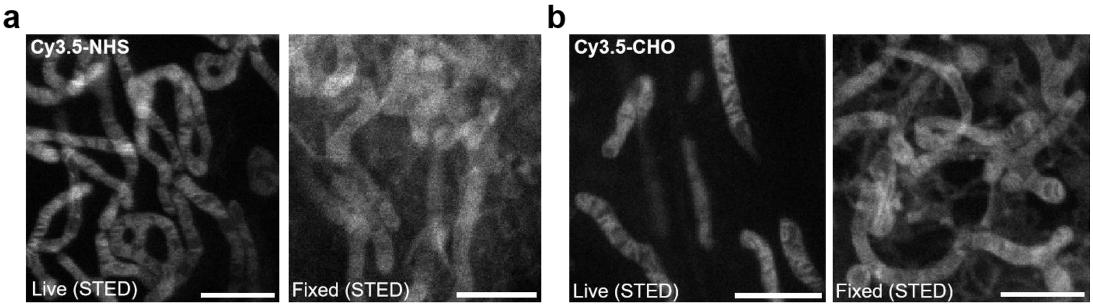

Fig S2. Cy3.5-NHS and Cy3.5-CHO are incompatible with post-fixation STED imaging.

a. STED images before (left) and after (right) fixation. HeLa cells were stained with 600 nM Cy3.5-NHS for 60 min before fixed with 2% GA and imaged at 10 min after fixation. Scale bars = 1 μm.

b. STED images before (left) and after (right) fixation. HeLa cells were stained with 1000 nM Cy3.5-CHO plus 8 μM verapamil for 60 min before fixed with 2% GA and imaged at 10 min after fixation. Scale bars = 1 μm.

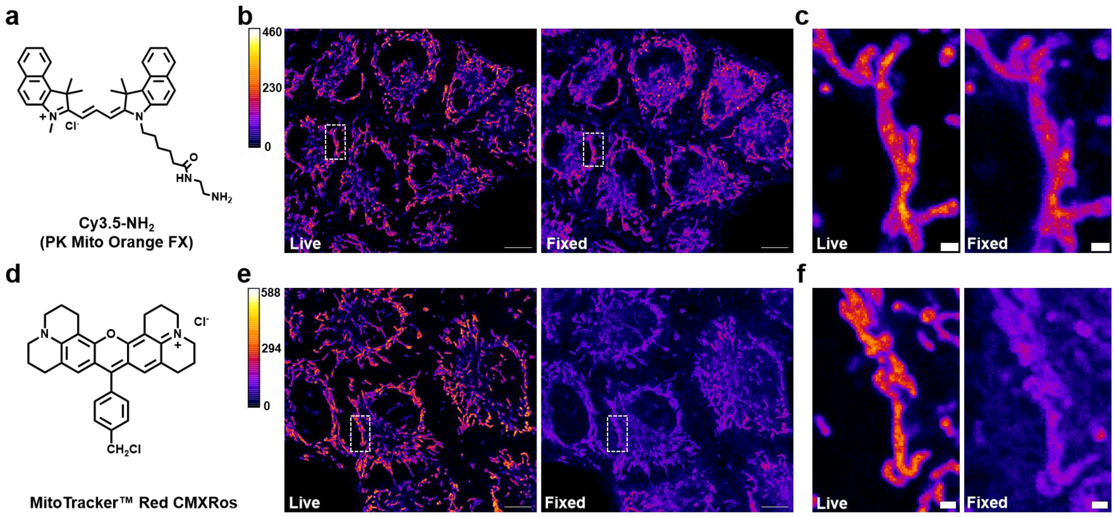

Fig S3. Comparison of signal to noise and fluorescence retention of Cy3.5-NH_2 and MitoTracker Red CMXRos (MTR) after fixation.

a. Chemical structure of Cy3.5-NH_2.

b. Confocal images from the same areas before (left) and after (right) fixation reflect the background raising and fluorescence retention of Cy3.5-NH_2. HeLa cells were stained with 600 nM Cy3.5-NH_2 plus 8 μM verapamil for 80 min before fixed with 2% GA and imaged at 10 min after fixation. Scale bars = 10 μm.

c. Zoomed-in image of mitochondria in the white dotted box area in figure b correspondingly. Scale bars = 1 μm.

d. Chemical structure of MTR.

e. Confocal images from the same areas before (left) and after (right) fixation reflect the background raising and fluorescence retention of MTR. HeLa cells were stained with 200 nM MTR for 20 min before fixed with 2% GA and imaged at 10 min after fixation. Scale bars = 10 μm.

f. Zoomed-in image of mitochondria in the white dotted box area in figure e correspondingly. Scale bars = 1 μm.

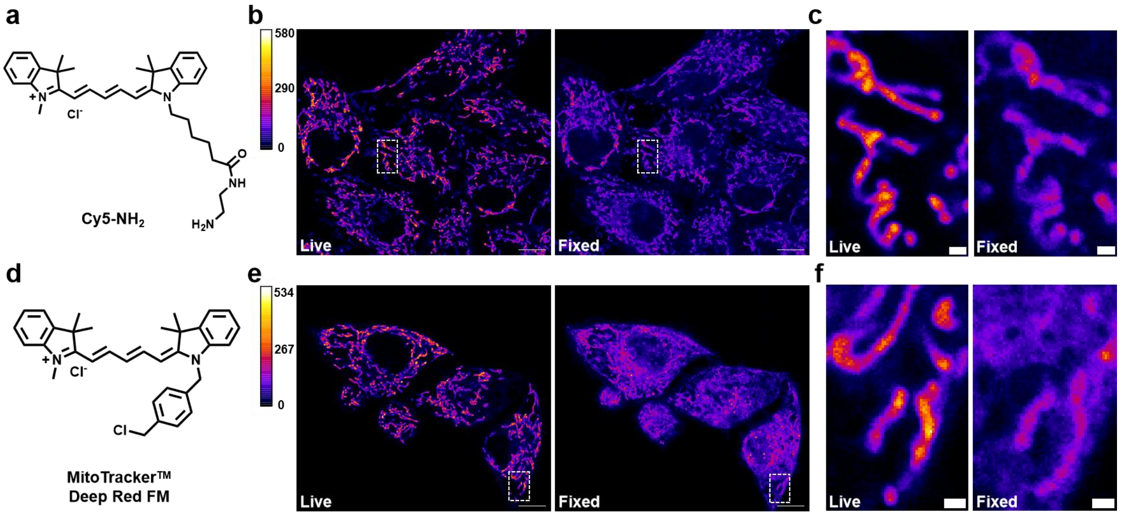

Fig S4. Comparison of signal to noise and fluorescence retention of Cy5-NH_2 and

MitoTracker Deep Red FM (MTDR) after fixation.

a. Chemical structure of Cy5-NH_2.

b. Confocal images from the same areas before (left) and after (right) fixation reflect the background raising and fluorescence retention of Cy5-NH_2. HeLa cells were stained with 500 nM Cy5-NH_2 plus 8 μM verapamil for 60 min before fixed with 2% GA and imaged at 10 min after fixation. Scale bars = 10 μm.

c. Zoomed-in image of mitochondria in the white dotted box area in figure b correspondingly. Scale bars = 1 μm.

d. Chemical structure of MTDR.

e. Confocal images from the same areas before (left) and after (right) fixation reflect the background raising and fluorescence retention of MTDR. HeLa cells were stained with 250 nM MTDR for 20 min before fixed with 2% GA and imaged at 10 min after fixation. Scale bars = 10 μm.

f. Zoomed-in image of mitochondria in the white dotted box area in figure e correspondingly. Scale bars = 1 μm.

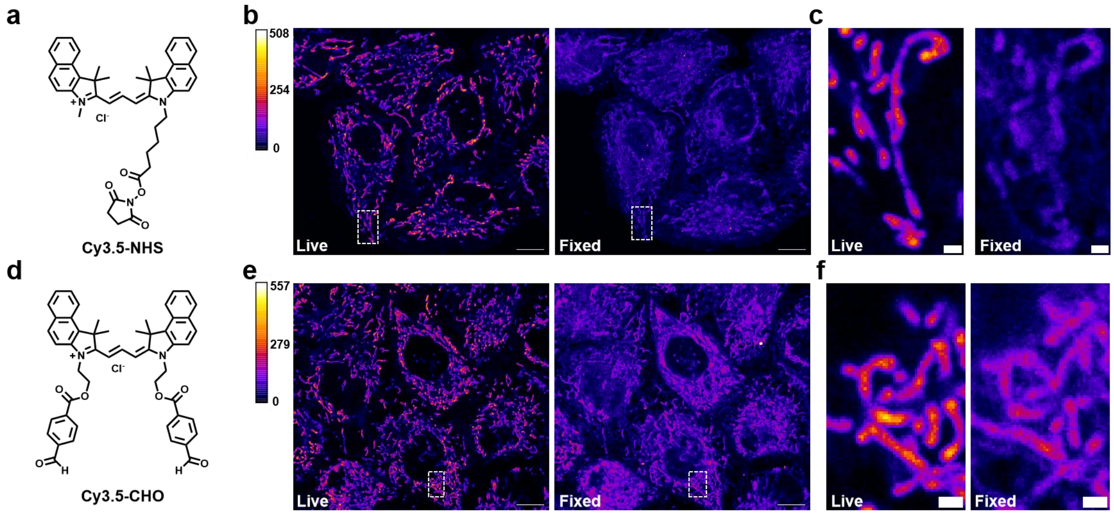

Fig S5. Comparison of signal to noise and fluorescence retention of Cy3.5-NHS and Cy3.5-CHO after fixation.

a. Chemical structure of Cy3.5-NHS.

b. Confocal images from the same areas before (left) and after (right) fixation reflect the background raising and fluorescence retention of Cy3.5-NHS. HeLa cells were stained with 600 nM Cy3.5-NHS plus 8 μM verapamil for 60 min before fixed with 2% GA and imaged at 10 min after fixation. Scale bars = 10 μm.

c. Zoomed-in image of mitochondria in the white dotted box area in figure b correspondingly. Scale bars = 1 μm.

d. Chemical structure of Cy3.5-CHO.

e. Confocal images from the same areas before (left) and after (right) fixation reflect the background raising and fluorescence retention of Cy3.5-CHO. HeLa cells were stained with 1 μM Cy3.5-CHO plus 8 μM verapamil for 60 min before fixed with 2% GA and imaged at 10 min after fixation. Scale bars = 10 μm.

f. Zoomed-in image of mitochondria in the white dotted box area in figure e correspondingly.

Scale bars = 1 μm.

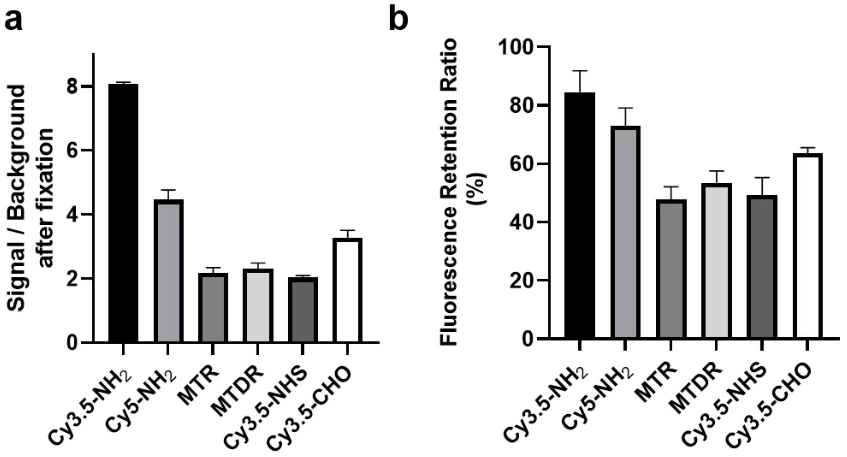

Fig S6. Histograms of Signal-to-Background ratio (SBR) and mitochondrial fluorescence retention ratio of six mitochondrial dyes after fixation.

a. Histograms of SBR of different mitochondrial dyes after fixation. SBR = Fluorescence intensity of the mitochondrial region / Fluorescence intensity in regions except mitochondria (background).

b. Histograms of MFR of different mitochondrial dyes after fixation. MFR = Fluorescence intensity of the mitochondrial region after fixation / Fluorescence intensity of the mitochondrial region before fixation.

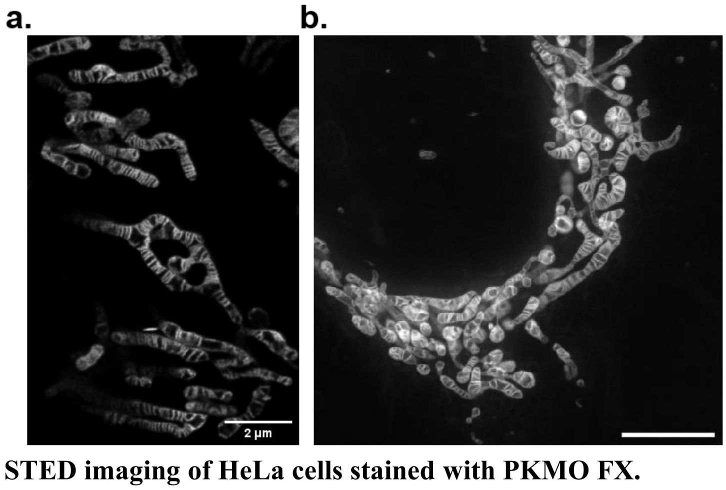

Fig S7. Live-cell STED imaging of HeLa cells stained with PKMO FX.

Cationic PKMO FX specifically accumulated in the IM of mitochondria and showed good performance when recorded with two different commercial STED nanoscopes.

a. STED recorded by STEDYCON, Abberior Instruments. Scale bar = 2 μm.

b. STED recorded by Leica TCS SP8 gSTED. Scale bar = 5 μm.

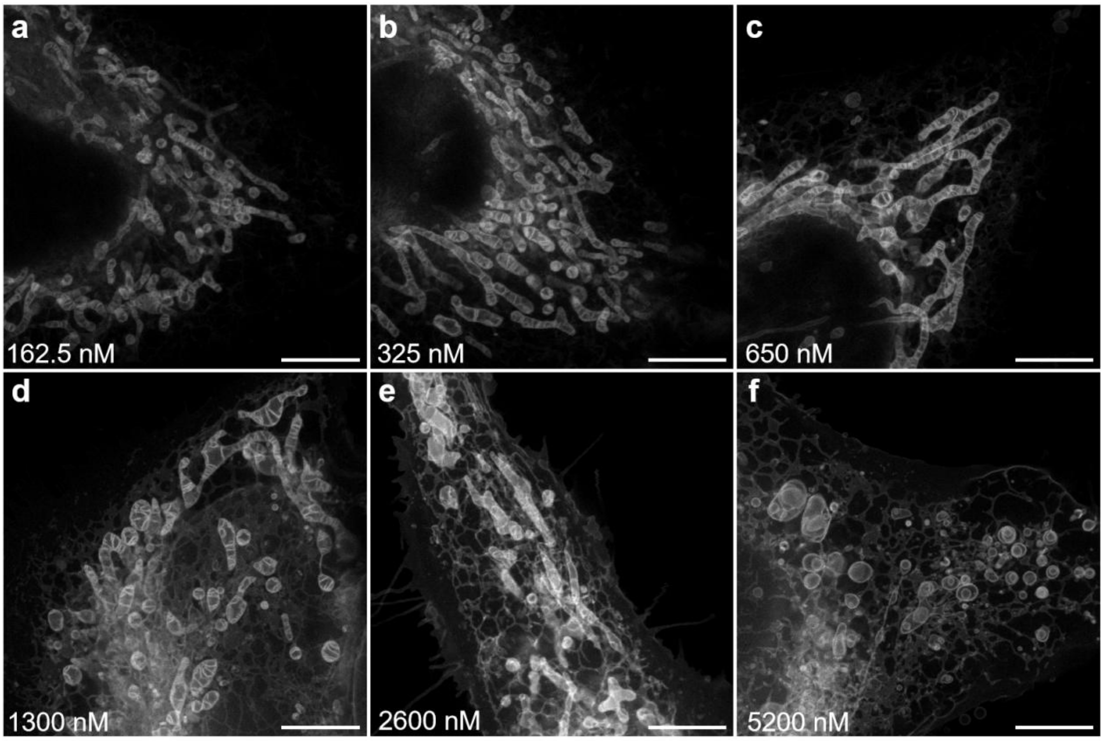

Fig S8. Comparison of mitochondrial morphology of HeLa cells stained with PKMO FX at different concentrations.

HeLa cells were stained with x nM PKMO FX plus 10 µM verapamil for 2 h. From a to f, x =162.5, 325, 650, 1300, 2600, and 5200 nM respectively. Concentrations exceeding 1300 nM were found to induce noticeable mitochondrial swelling and roundness. Therefore, we recommend depending on the used cell line a staining concentration between 150-700 nM, an incubation time between 15 min and 2 h and optionally the addition of verapamil.

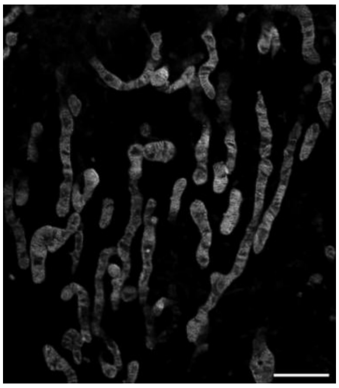

Fig S9. STED image of a HeLa cell stained with PKMO FX, 13 days after staining and fixation.

PKMO FX offers long-term and stable cross-linking which enables STED imaging over 10 days after fixation. HeLa cells were labeled with PKMO FX and then fixed with 2% GA for 15 min. Cells are washed with ddH_2O and stored under 4°C before repeatedly imaged. The mitochondrial cristae are reproducibly visible with STED nanoscopy after 13 days. Scale bar = 2 µm.

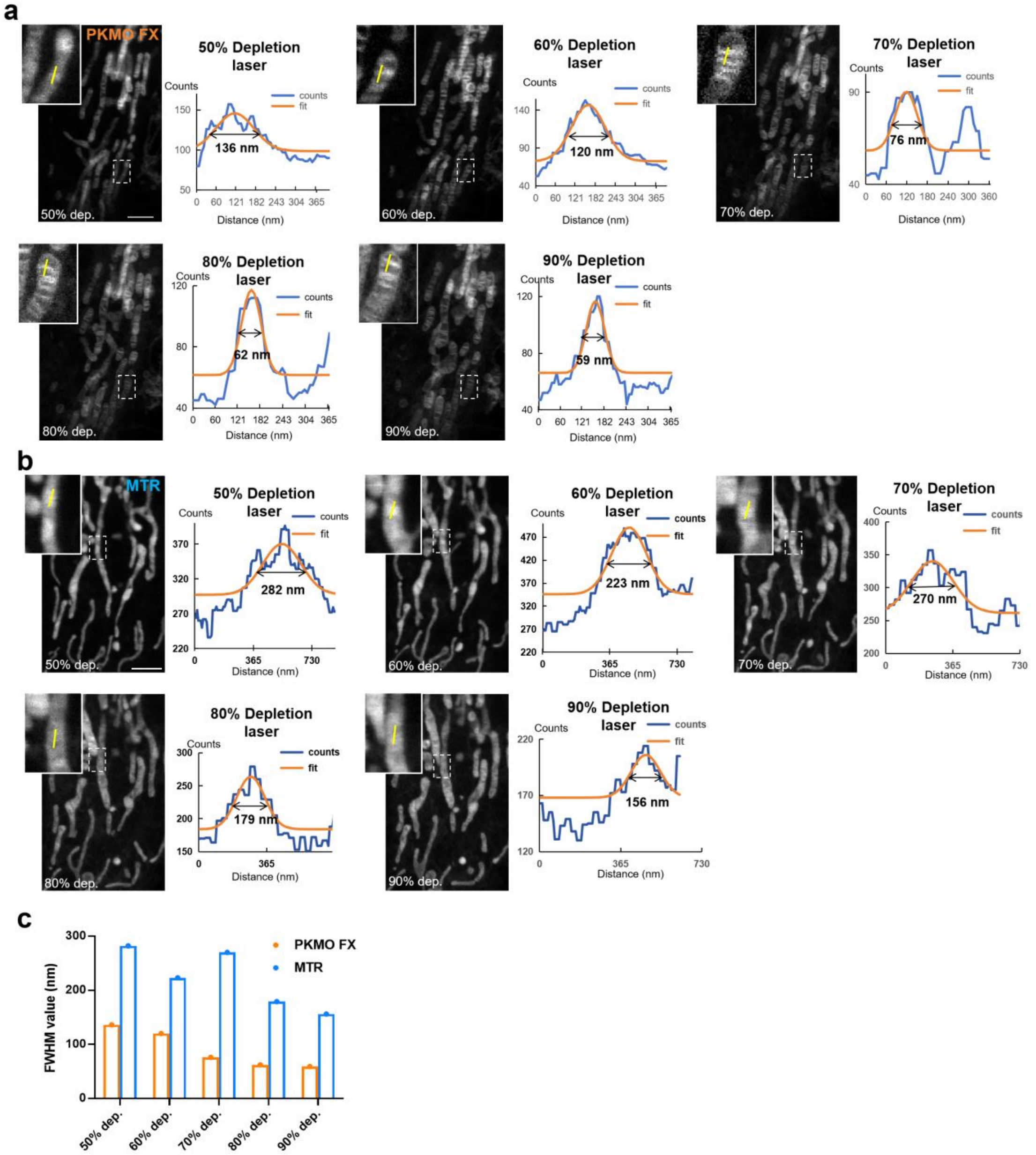

Fig S10. Live-cell STED imaging under different depletion laser powers of HeLa cells stained with PKMO FX or MTR.

a. Raw STED images and analysis of FWHM of fluorescence intensity line profiles recorded from HeLa cells stained with PKMO FX. HeLa cells were stained with 600 nM PKMO FX for 60 min, then imaged using different depletion laser powers from 50% to 90%, respectively. Fluorescence intensity line profiles measured across mitochondrial cristae (from the yellow line area in the white dotted box) are shown on the right of each image. Zoomed-in images of the mitochondria from the yellow line area are shown on the upper left of each image. The FWHM was calculated from a Gaussian fitting within the STEDYCON software, Abberior Instruments. Scale bars = 2 μm.

b. Raw STED images and analysis of FWHM of fluorescence intensity line profiles recorded from HeLa cells stained with MTR. HeLa cells were stained with 250 nM MTR for 30 min, then imaged using different depletion laser powers from 50% to 90%, respectively. Fluorescence

intensity line profiles measured across mitochondrial cristae (from the yellow line area in the white dotted box) are shown on the right of each image. Zoomed-in images of the mitochondria from the yellow line area are shown on the upper left of each image. The FWHM was calculated from a Gaussian fitting within the STEDYCON software, Abberior Instruments. Scale bars = 2 µm.

c. Histogram of FWHM values of the two mitochondrial dyes under different depletion power.

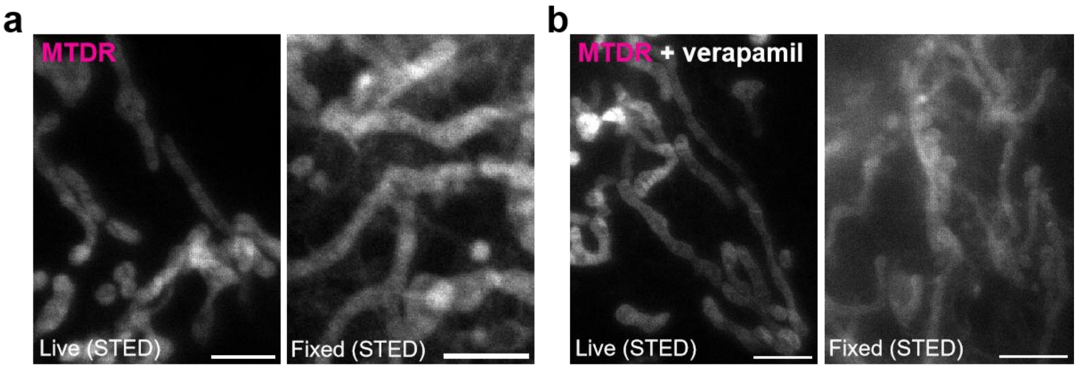

Fig S11. MTDR is not suitable for both live-cell and post-fixation STED imaging.

a. Raw STED images of HeLa cells stained with MTDR in the absence of verapamil. HeLa cells were stained with 200 nM MTDR for 20 min and fixed with 2.5% GA. Images were taken before fixation and 10 min after fixation. Scale bars = 2 µm.

b. Raw STED images of HeLa cells stained with MTDR in the presence of verapamil. HeLa cells were stained with 500 nM MTDR plus 8 µM verapamil for 60 min and fixed with 2.5% GA. Images were taken before fixation and 10 min after fixation. Scale bars = 2 µm.

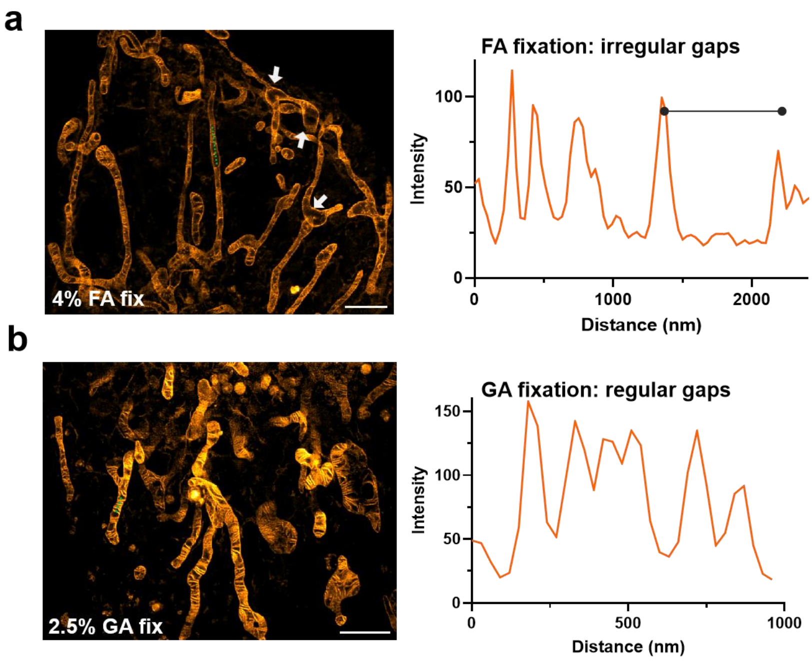

Figure S12. Comparison of FA/GA fixatives on mitochondrial morphology.

a. FA fixation can cause significant swelling and roundedness (highlighted with white arrow) in

some mitochondria which was less frequent in GA-fixed cells. Scale bar = 2 μm. Plot profile from the green line showed noticeable irregular crista-to-crista spacing brought by FA fixation.

b. GA fixation can bring more normal mitochondrial morphology. Scale bar = 2 μm. Plot profile from the green line showed mostly regular crista-to-crista spacing.

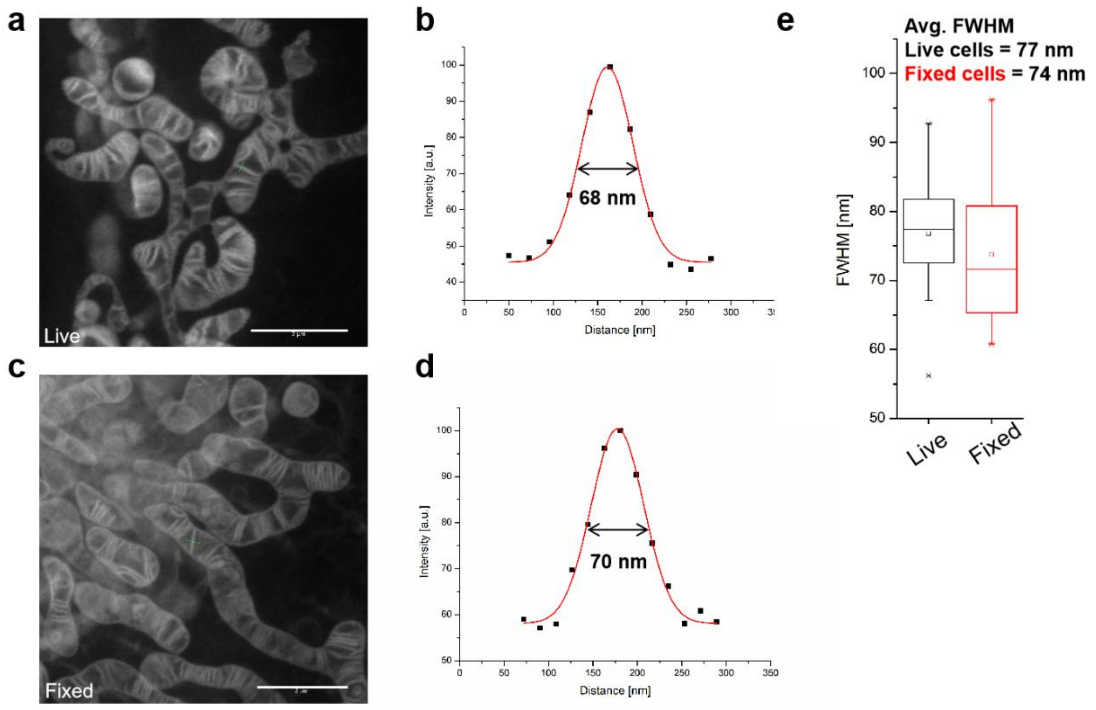

Fig S13. Resolution estimation of nanoscopic mitochondrial images.

a. Raw data of the STED image of a live HeLa cell. Scale bar = 2μm.

b. Fluorescence intensity line profile as indicated by the green line area in a. The fluorescence intensity signal was fitted using a Gaussian fit (red line). The full width at half maximum (FWHM) value was estimated for the individual peak and calculated as 68 nm.

c. Raw data of the STED image of a fixed HeLa cell. Scale bar = 2μm.

d. Fluorescence intensity line profile as indicated by the green line area in c. The fluorescence intensity signal was fitted using a Gaussian fit (red line). The FWHM value was estimated for the individual peak and calculated as 70 nm.

e. Box plot graphs of the FWHM statistics of the live cells and fixed cells. 19 individual cristae from 3 STED images of live cells and 19 individual cristae from 3 STED images of fixed cells were used. The average FWHM of live HeLa cells was 77 nm, and the average FWHM of fixed HeLa cells was 74 nm.

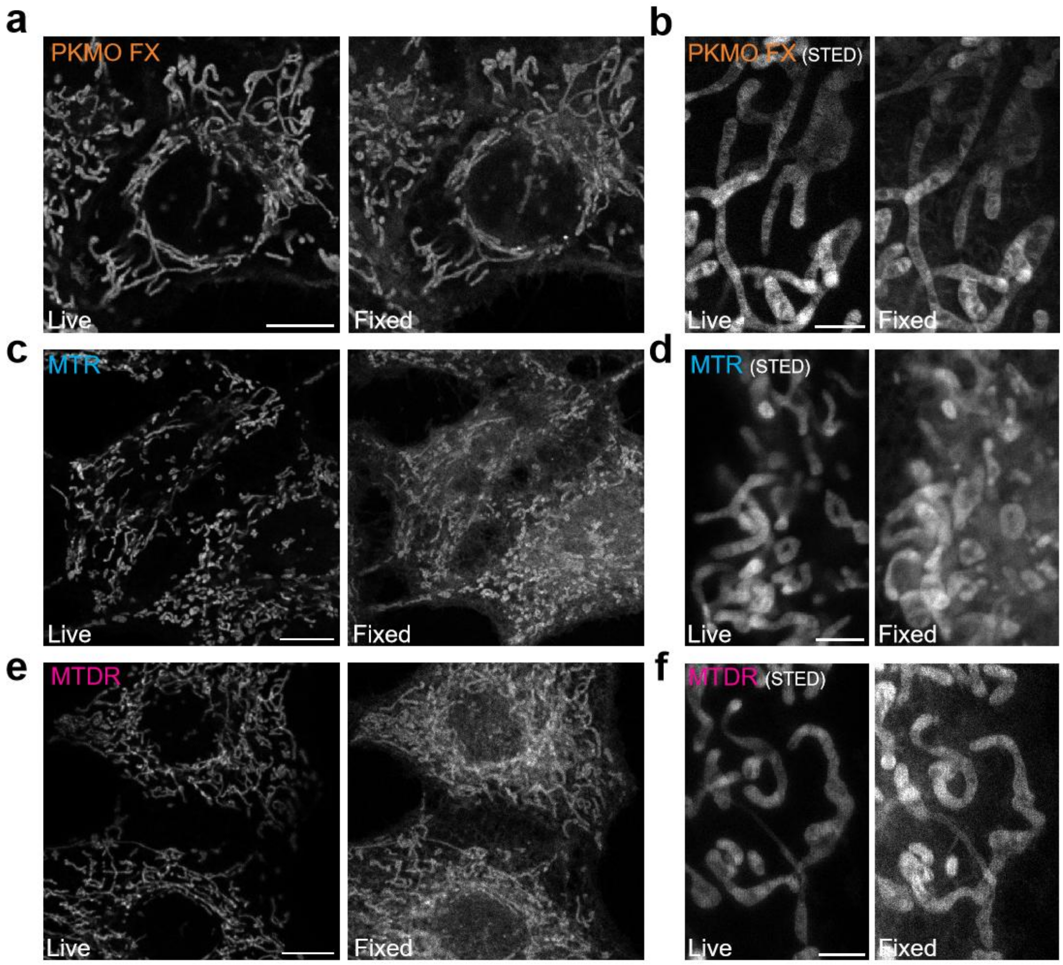

g

Dyes	PKMO FX	MTR	MTDR
Staining condition	200 nM, 15 min		
Fixation condition	4% FA + 0.1% GA, 37°C, 10 min		
SBR after fixation	5.5 ± 0.3	1.9 ± 0.3	2.2 ± 0.4

Fig S14. Comparison of SBR and STED imaging performance of PKMO FX, MTR and MTDR after fixation by 4% FA plus 0.1% GA.

a. Confocal images of HeLa cells stained with PKMO FX. Images of same area were taken before and after fixation. Scale bars = 10 μm.

b. STED images of HeLa cells stained with PKMO FX. Images of same area were taken before and after fixation. Scale bars = 2 μm.

c. Confocal images of HeLa cells stained with MTR. Images of same area were taken before and after fixation. Scale bars = 10 μm.

d. STED images of HeLa cells stained with MTR. Images of same area were taken before and after fixation. Scale bars = 2 μm.

e. Confocal images of HeLa cells stained with MTDR. Images of same area were taken before and after fixation. Scale bars = 10 μm.

f. STED images of HeLa cells stained with MTDR. Images of same area were taken before and after fixation. Scale bars = 2 μm.

g. Staining and fixation conditions and the statistical SBR of the three mitochondrial dyes.

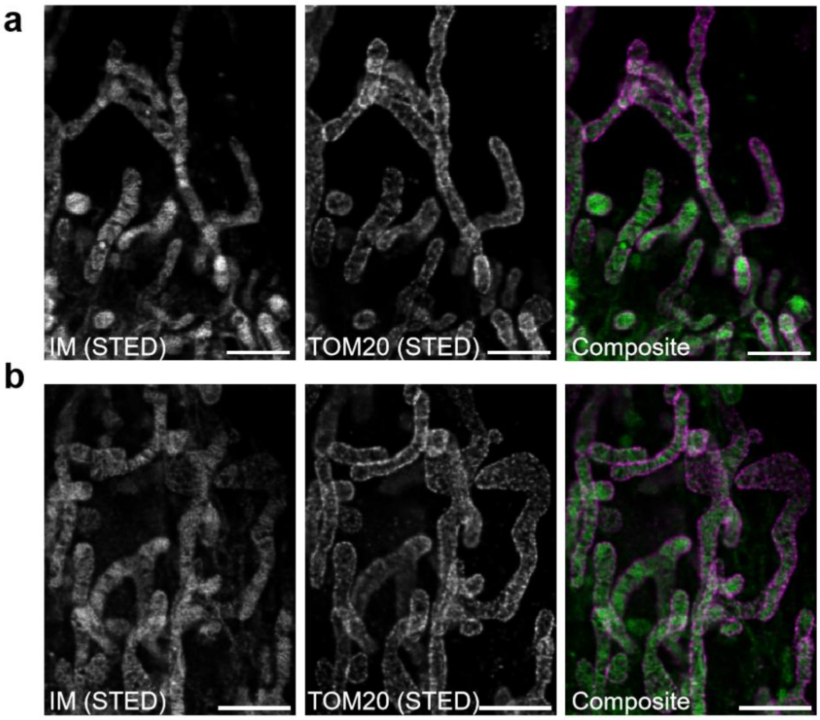

Fig S15. Immunolabeling of HeLa cells fixed by 4% FA plus 0.1% GA. The cristae were visible but not as good as those obtained under sequential fixation condition.

a,b: STED imaging of IM and OM from two different views. HeLa cells were first stained with PKMO FX (500 nM, 1 h) and then fixed with 4% FA plus 0.1% GA (37 °C) for 10 min before subsequent immunolabeling of Tom20. From left to right: split channels of IM, OM and composite. Scale bars = 2 μm.

Chemical synthesis and characterization

General experimental information

Unless otherwise mentioned, all the chemicals were purchased at the highest commercial quality and used without further purification unless otherwise stated.

Reactions were monitored by Thin Layer Chromatography (TLC) on plates (GF254) supplied by Yantai Chemicals (China) using UV light as a visualizing agent or by LC/MS (4.6 mm ×150 mm 5 μm C18 column; 5 μL injection; 10-100% CH_3CN/H_2O, linear-gradient, with constant 0.1% v/v TFA additive; 30 min run; 1 mL/min flow; ESI; positive ion mode; UV detection at 254 nm). Flash column chromatography was performed with silica gel (200-300 mesh) supplied by Tsingtao Haiyang Chemicals (China).

NMR spectra were recorded on Brüker Advance 400 (^1H 400 MHz, ^{13}C 101 MHz) and are calibrated using residual solvent (DMSO-d_6 at 2.50 ppm ^1H NMR, $CDCl_3$ at 7.26 ppm ^1H NMR). Data for ^1H NMR spectra are reported as follows: chemical shift (δ ppm), multiplicity (s = singlet, d = doublet, t = triplet, q = quartet, dd = doublet of doublets, dt = triplet of doublets, m=multiplet, br=broad), coupling constant (Hz), integration. Data for ^{13}C NMR are reported by chemical shift (δ ppm).

Synthesis of Cy3.5-NH_2 / PK Mito Orange FX

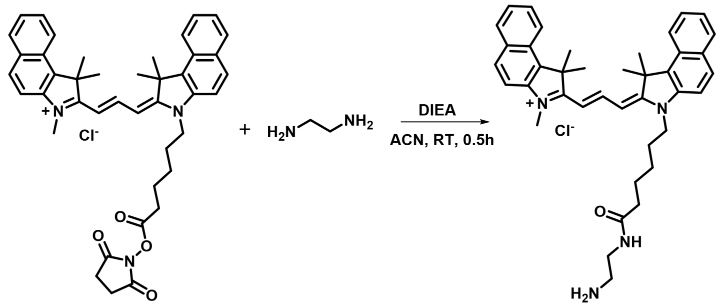

To a solution of Cyanine3.5 NHS ester (5.0 mg, 7.2 μmol, 1.0 *eq*, CONFLUORE, China) in acetonitrile (0.4 mL) was added ethylenediamine (8.7 mg, 10 μL, 144 μmol, 20 *eq*) and N, N-Diisopropylethylamine (18 mg, 10 μL, 72 μmol, 10 *eq*). The mixture was stirred at 25 °C for 0.5 h. TLC showed the starting material was consumed completely. The solvent was removed *in vacuo* and the crude product was purified by reversed-phase High Performance Liquid Chromatography (HPLC, eluent A: ddH_2O containing 0.1% TFA (v/v); eluent B: CH_3CN. A 30-min linear gradient, from 10% to 100% solvent B; flow rate: 10 mL/min; detection wavelength: 254 nm and 590 nm). The solvent was removed via rotary evaporation to give the product Cy3.5-NH_2 (5.9 μmol, 82% yield) as deep purple solid. The amount of Cy3.5-NH_2 was determined based on the absorbance at 584 nm ($\varepsilon_{MeOH} = 1.05\times10^5$ $M^{-1}cm^{-1}$)(4).

^1H NMR (400 MHz, DMSO-d_6) δ 8.58 (t, J = 13.5 Hz, 1H), 8.29 (dd, J = 8.6, 3.2 Hz, 2H), 8.15 – 8.03 (m, 4H), 7.90 (s, 1H), 7.79 (dd, J = 10.8, 8.9 Hz, 2H), 7.69 (ddt, J = 8.4, 6.8, 1.6 Hz, 2H), 7.54 (ddt, J = 8.2, 6.8, 1.3 Hz, 2H), 6.56 (d, J = 13.5 Hz, 2H), 4.24 (t, J = 6.4 Hz, 2H), 3.80 (s, 3H), 3.26 (q, J = 6.2 Hz, 2H), 2.84 (q, J = 6.2 Hz, 2H), 2.12 (d, J = 7.4 Hz, 2H), 2.01 (d, J = 2.3 Hz, 12H), 1.85 – 1.75 (m, 2H), 1.65 – 1.57 (m, 2H), 1.50 – 1.41 (m, 2H), 0.83 (m, 2H).

^{13}C NMR (101 MHz, DMSO-d_6) δ 175.73, 174.91, 172.78, 157.84, 157.82, 148.36, 140.28, 139.59, 133.03, 133.01, 131.54, 131.46, 130.47, 130.37, 129.96, 127.88, 127.45, 127.37, 125.03, 122.20, 122.16, 121.79, 111.74, 111.68, 102.54, 101.86, 79.31, 78.97, 78.64, 50.58, 50.55, 43.44,

38.63, 36.37, 35.12, 27.11, 26.92, 25.85, 24.78, 22.09.

MS (ESI) calculated for $C_{40}H_{47}N_4O^+$ (M^+ 599.8) , observed (M^+ 599.4)

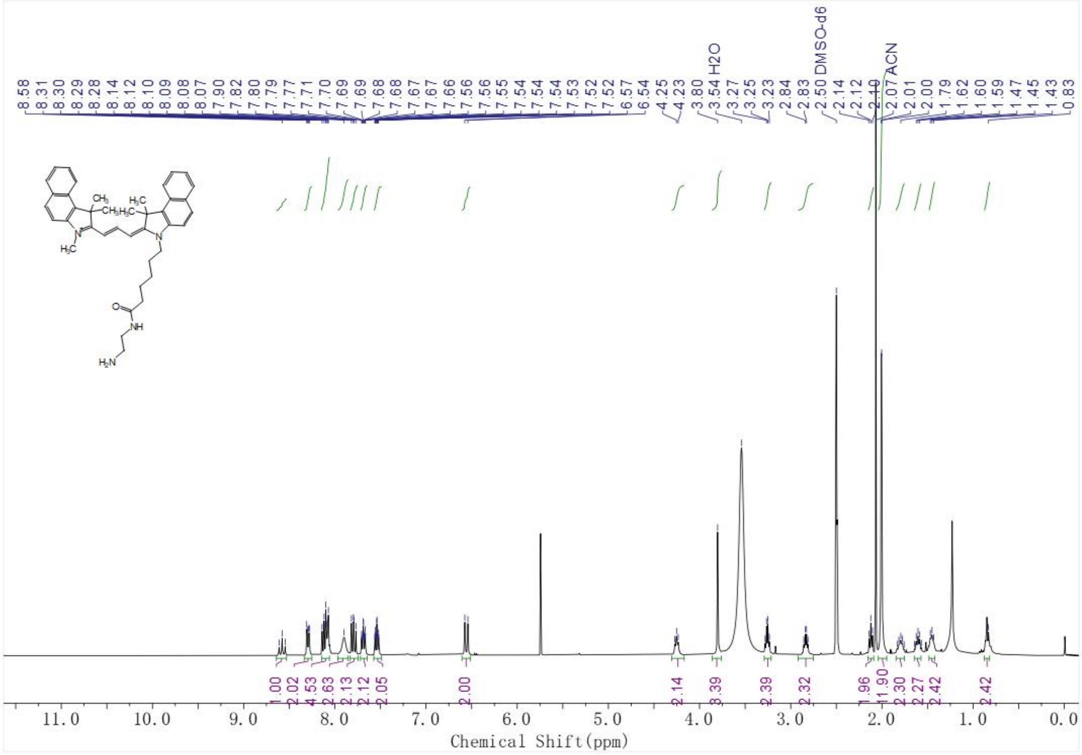

^{1}H NMR spectrum of Cy3.5-NH$_2$ / PK Mito Orange FX

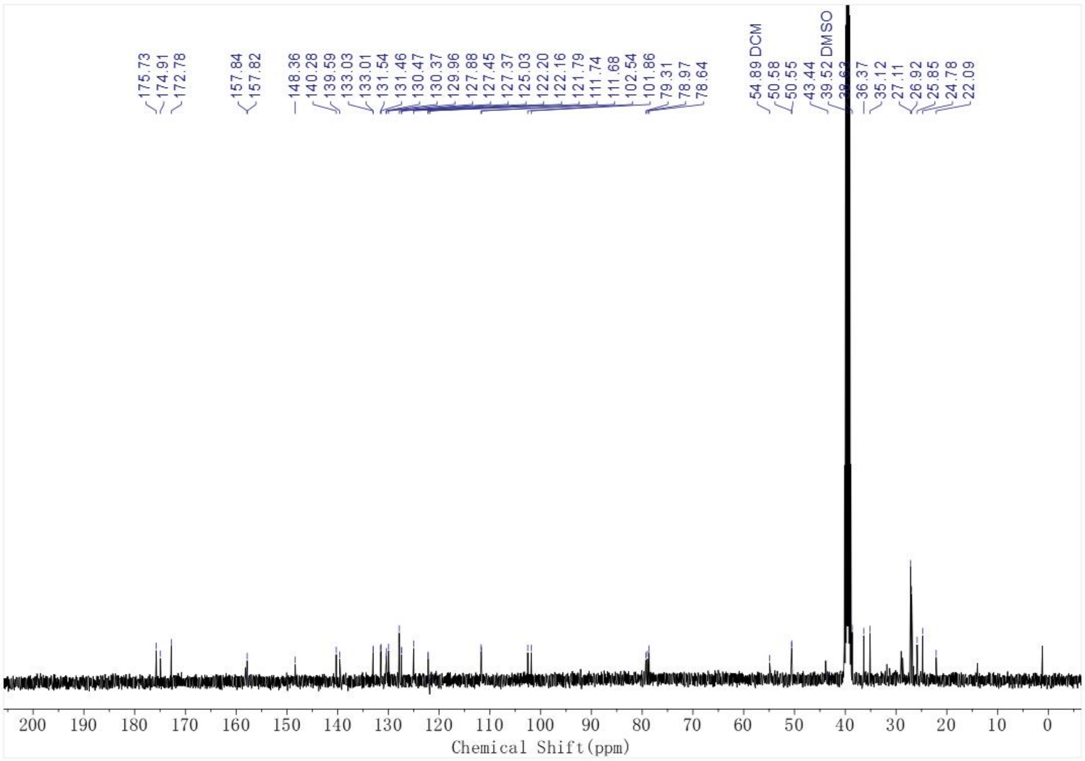

^{13}C NMR spectrum of Cy3.5-NH$_2$ / PK Mito Orange FX

Synthesis of Cy5-NH_2

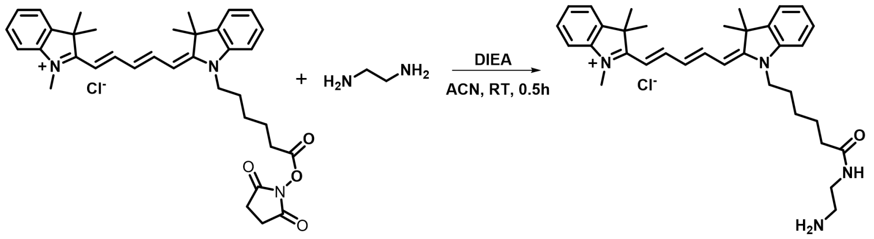

To a solution of Cyanine5 NHS ester (5.0 mg, 8.1 μmol, 1.0 *eq*, CONFLUORE, China) in acetonitrile (0.4 mL) was added ethylenediamine (9.8 mg, 11 μL, 162 μmol, 20 *eq*) and N, N-Diisopropylethylamine (20 mg, 11 μL, 81 μmol, 10 *eq*). The mixture was stirred at 25 °C for 0.5 h. TLC showed the starting material was consumed completely. The solvent was removed *in vacuo* and the crude product was purified by reversed-phase HPLC (eluent A: ddH_2O containing 0.1% TFA (v/v); eluent B: CH_3CN. A 30-min linear gradient, from 10% to 100% solvent B; flow rate: 10 mL/min; detection wavelength: 254 nm and 640 nm). The solvent was removed via rotary evaporation to give the product Cy5-NH_2 (6.3 μmol, 77% yield) as deep blue solid. The amount of Cy5-NH_2 was determined based on the absorbance at 646 nm ($\varepsilon_{\mathrm{MeOH}} = 2.5\times10^5$ $M^{-1}cm^{-1}$).

^{1}H NMR (400 MHz, DMSO-d_6) δ 8.33 (t, J = 13.1 Hz, 2H), 7.99 (t, J = 5.2 Hz, 2H), 7.81 (s, 1H), 7.62 (d, J = 7.4 Hz, 2H), 7.43-7.37 (m, 2H), 7.25 (ddd, J = 12.0, 6.4, 1.9 Hz, 2H), 6.56 (t, J = 12.4 Hz, 1H), 6.28 (dd, J = 16.7, 13.8 Hz, 2H), 4.08 (t, J = 7.4 Hz, 2H), 3.60 (s, 3H), 3.24 (q, J = 6.3 Hz, 2H), 2.82 (q, J = 6.3 Hz, 2H), 2.09 (t, J = 7.4 Hz, 2H), 1.68-1.64 (m, 14H), 1.57-1.53 (m, 2H), 1.37-1.33 (m, 2H), 1.23 (s, 2H).

MS (ESI) calculated for $C_{34}H_{45}N_4O^+$ (M^+ 525.7) , observed (M^+ 525.5)

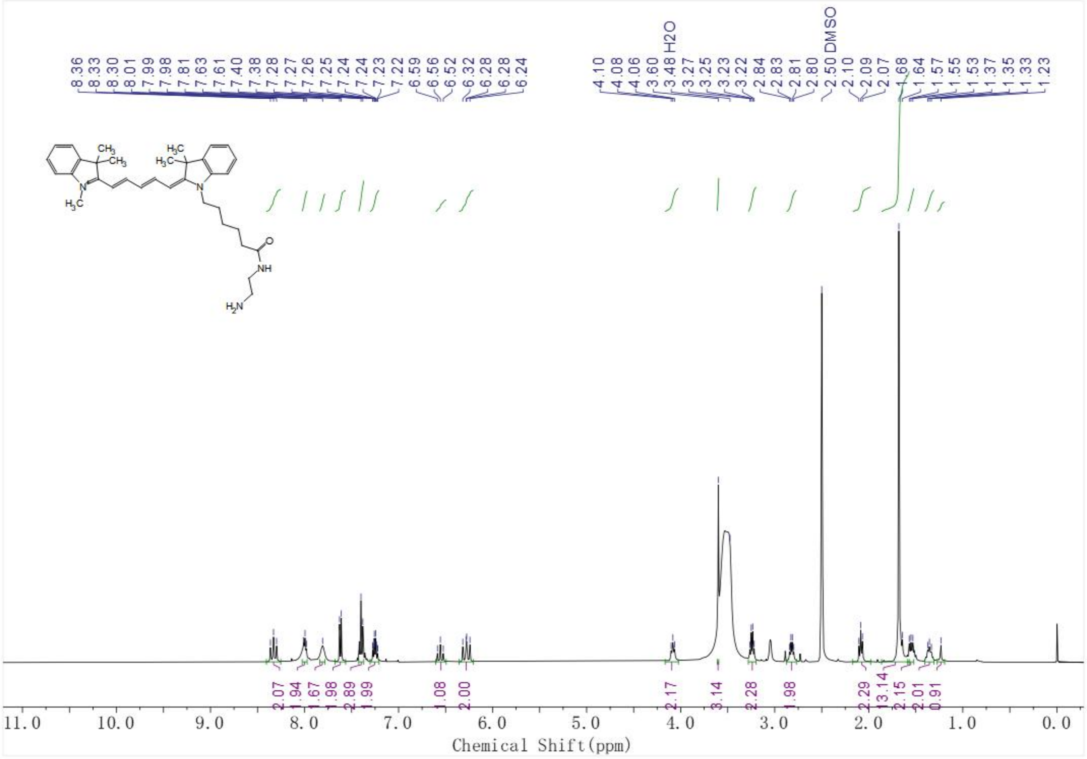

^{1}H NMR spectrum of Cy5-NH_2

Synthesis of Cy3.5-CHO

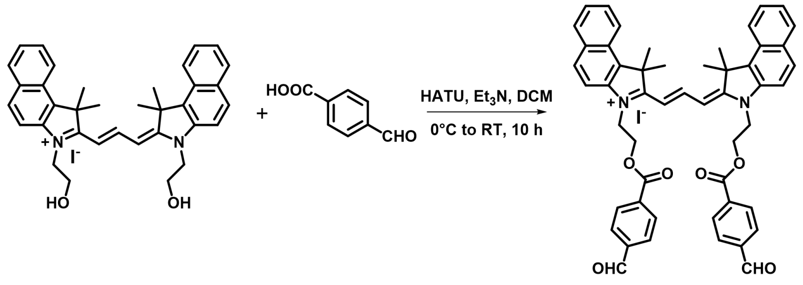

1 **Cy3.5-CHO**

Compound **1** was synthesized according to the method reported in a previous reference (5). Compound **1** (3.5 mg, 5 µmol, 1.0 *eq*) was dissolved in dichloromethane (1 mL) and cooled to 0℃ in an ice bath. The 4-carboxybenzaldehyde (2.4 mg, 16 µmol, 3.2 *eq*) was dissolved in dichloromethane (2 mL) in which 2-(7-Azabenzotriazol-1-yl)-N,N,N',N'-tetramethyluronium hexafluorophosphate (HATU, 8.2 mg, 21 µmol, 4.2 *eq*) and triethylamine (3 µL, 21 µmol, 4.2 *eq*) were added. The mixture was stirred in an ice bath for 30 min, then the solution of compound **1** in dichloromethane was added slowly into the mixture. The mixture was stirred at room temperature for 10 h before the solvent was removed *in vacuo*. The residue was purified by silica column chromatography (dichloromethane: methanol =100:1) to obtain Cy3.5-CHO (0.70 µmol, 14% yield) as deep purple solid.

^{1}H NMR (400 MHz, $CDCl_3$) δ 9.96 (s, 2H), 8.57 (t, J = 13.1 Hz, 1H), 8.07 (d, J = 8.5 Hz, 2H), 7.96 (d, J = 7.8 Hz, 4H), 7.90 (d, J = 7.2 Hz, 2H), 7.84 (d, J = 8.6 Hz, 2H), 7.79 (d, J = 7.7 Hz, 4H), 7.61 (t, J = 7.2 Hz, 2H), 7.53-7.44 (m, 4H), 6.87 (d, J = 13.1 Hz, 2H), 4.43 (t, J = 5.2 Hz, 4H), 3.40-3.37 (m, 4H), 1.97 (s, 12H).

MS (ESI) calculated for $C_{51}H_{45}N_2O_6^+$ (M^+ 781.3) , observed (M^+ 781.4)

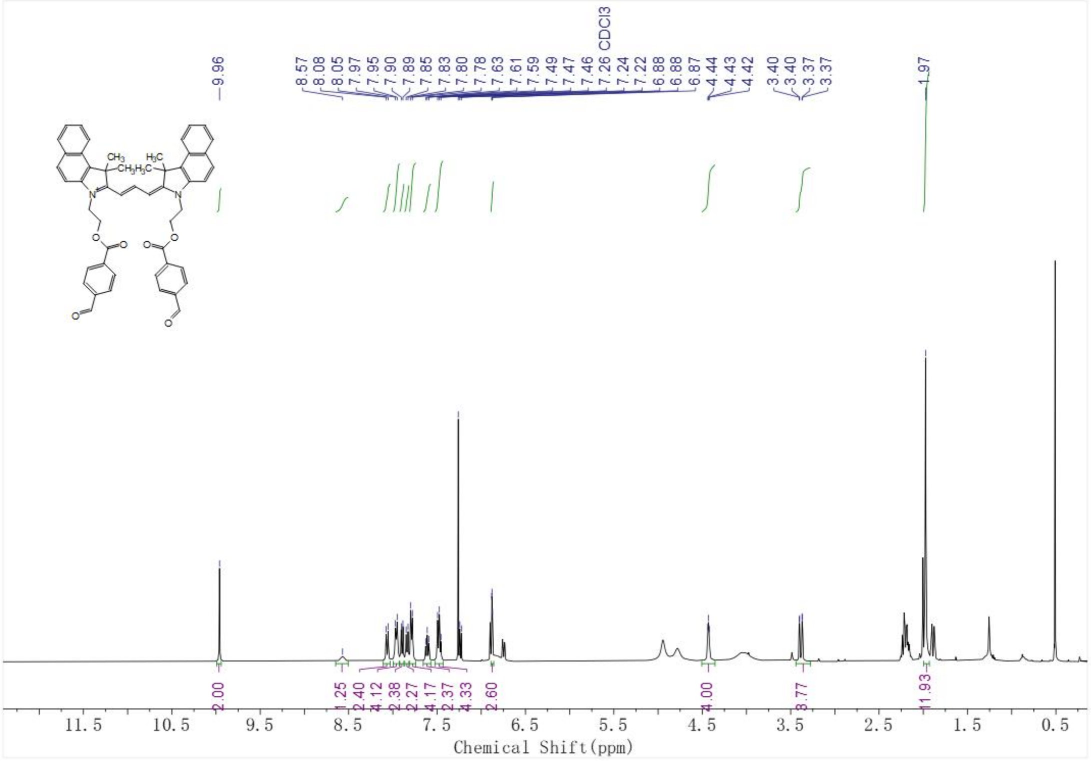

^{1}H NMR spectrum of Cy3.5-CHO

Reference

1. Mastronarde DN (2005) Automated electron microscope tomography using robust prediction of specimen movements. *Journal of Structural Biology* 152(1):36-51.
2. Kremer JR, Mastronarde DN, & McIntosh JR (1996) Computer Visualization of Three-Dimensional Image Data Using IMOD. *Journal of Structural Biology* 116(1):71-76.
3. Heiligenstein X, Paul-Gilloteaux P, Raposo G, & Salamero J (2017) Chapter 16 - eC-CLEM: A multidimension, multimodel software to correlate intermodal images with a focus on light and electron microscopy. *Methods in Cell Biology*, eds Müller-Reichert T & Verkade P (Academic Press), Vol 140, pp 335-352.
4. Lopalco M, Koini EN, Cho JK, & Bradley M (2009) Catch and release microwave mediated synthesis of cyanine dyes. *Organic & Biomolecular Chemistry* 7(5):856-859.
5. Liu T, *et al.* (2022) Multi-color live-cell STED nanoscopy of mitochondria with a gentle inner membrane stain. *Proceedings of the National Academy of Sciences* 119(52):e2215799119.
